# UNC-30/PITX coordinates neurotransmitter identity with postsynaptic GABA receptor clustering

**DOI:** 10.1242/dev.202733

**Published:** 2024-08-27

**Authors:** Edgar Correa, Morgane Mialon, Mélissa Cizeron, Jean-Louis Bessereau, Berangere Pinan-Lucarre, Paschalis Kratsios

**Affiliations:** ^1^ Department of Neurobiology, University of Chicago, Chicago, IL 60637, USA; ^2^ Committee on Cell and Molecular Biology, University of Chicago, Chicago, IL 60637, USA; ^3^ Melis, Universite Claude Bernard Lyon 1, CNRS UMR5284, INSERM U1314, Institut NeuroMyoGene - Faculte de Medecine et de Pharmacie, 69008 Lyon, France; ^4^ University of Chicago Neuroscience Institute, Chicago, IL 60637, USA

**Keywords:** Neuronal connectivity, Terminal selectors, UNC-30/PITX, MADD-4/ADAMTSL, GABA receptors, *C*. *elegans*

## Abstract

Terminal selectors are transcription factors that control neuronal identity by regulating expression of key effector molecules, such as neurotransmitter biosynthesis proteins and ion channels. Whether and how terminal selectors control neuronal connectivity is poorly understood. Here, we report that UNC-30 (PITX2/3), the terminal selector of GABA nerve cord motor neurons in *Caenorhabditis elegans*, is required for neurotransmitter receptor clustering, a hallmark of postsynaptic differentiation. Animals lacking *unc-30* or *madd-4B*, the short isoform of the motor neuron-secreted synapse organizer *madd-4* (punctin*/ADAMTSL*), display severe GABA receptor type A (GABA_A_R) clustering defects in postsynaptic muscle cells. Mechanistically, UNC-30 acts directly to induce and maintain transcription of *madd-4B* and GABA biosynthesis genes (e.g. *unc-25/GAD*, *unc-47/VGAT*). Hence, UNC-30 controls GABA_A_ receptor clustering in postsynaptic muscle cells and GABA biosynthesis in presynaptic cells, transcriptionally coordinating two crucial processes for GABA neurotransmission. Further, we uncover multiple target genes and a dual role for UNC-30 as both an activator and a repressor of gene transcription. Our findings on UNC-30 function may contribute to our molecular understanding of human conditions, such as Axenfeld–Rieger syndrome, caused by *PITX2* and *PITX3* gene variants.

## Teaser

**Summary:** In *C. elegans* motor neurons, a PRD-class homeodomain transcription factor coordinates neurotransmitter biosynthesis in the presynaptic cell with clustering of cognate neurotransmitter receptors at the postsynaptic cell.

## INTRODUCTION

In the nervous system, neuronal communication depends on the proper transmission of signals through chemical and electrical synapses. In the context of chemical synapses, presynaptic neurons must be able to synthesize and package into synaptic vesicles specific chemical substances known as neurotransmitters (NTs), such as acetylcholine (ACh), gamma-aminobutyric acid (GABA) and glutamate (Glu). Upon secretion into the synaptic cleft, each NT molecule binds to its cognate receptor located at the postsynaptic cell membrane, thereby evoking postsynaptic electrical responses.

Genes encoding proteins for NT biosynthesis and packaging (e.g. enzymes, transporters) are co-expressed in specific neuron types. The co-expression of these proteins defines the NT identity (or NT phenotype) of individual neuron types (e.g. cholinergic, GABAergic, dopaminergic). Although instances of NT identity switching or multi-NT use by single neurons have been described (Dulcis et al., 2013; Pereira et al., 2015; Brunet Avalos and Sprecher, 2021; Pedroni and Ampatzis, 2019), it is generally the case that individual neuron types acquire a specific NT identity during development and maintain it throughout life, consistent with Dale's principle of ‘one neuron, one NT’ (Svensson et al., 2019). The continuous expression of NT identity genes is fundamental for the ability of a presynaptic neuron to signal to its postsynaptic targets. For efficient neurotransmission, however, it is equally important that cognate NT receptors cluster at postsynaptic domains precisely juxtaposed to presynaptic boutons (Gravielle, 2021; Mizumoto et al., 2023). Whether and how these two crucial processes, i.e. NT identity of the presynaptic neuron and NT receptor clustering at the postsynaptic cell, are coordinated remains poorly understood.

Genetic studies in nematodes (*Caenorhabditis elegans*), fruit flies (*Drosophila melanogaster*) and mice have revealed a phylogenetically conserved principle for the control of NT identity: transcription factors expressed in specific neuron types, termed ‘terminal selectors’, coordinate the expression of NT identity genes, thereby coordinating synthesis of enzymes and transporters necessary for NT biosynthesis and signaling (Hobert, 2008; Hobert and Kratsios, 2019). Terminal selectors broadly control batteries of genes encoding proteins essential for neuronal identity and function (e.g. ion channels, neuropeptides) (Hobert, 2011; Hobert and Kratsios, 2019). To date, terminal selectors have been predicted for 117 of the 118 *C. elegans* neuron types (Hobert, 2016; Reilly et al., 2022; Glenwinkel et al., 2021). Beyond *C. elegans*, terminal selectors have also been identified in *D. melanogaster*, cnidarians (*Nematostella vectensis*), marine chordates (*Ciona intestinalis*) and mice (*Mus musculus*) (Hobert and Kratsios, 2019), suggesting a deeply conserved role for these crucial regulators of NT identity. A defining feature of terminal selectors is their continuous expression – from development throughout adulthood – in specific neuron types (Hobert, 2008). Although the essential roles of terminal selectors in establishing NT identity during development are well-attested across model organisms, their involvement in maintaining NT identity in later life stages remains poorly examined (Achim et al., 2014), partially owing to the lack of genetic tools for inducible terminal selector depletion in late life stages.

**Fig. 1.**
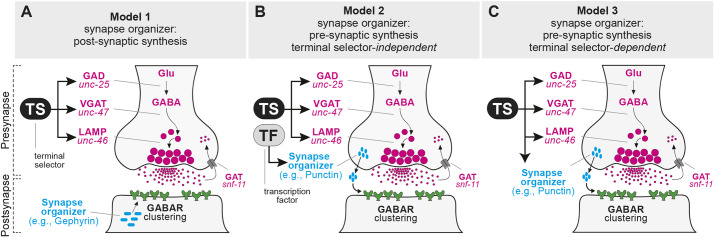
**Hypothetical models for transcriptional control of GABA synapse organizers.** (A) Model 1: A synapse organizer is produced in the target cell and controls GABAR clustering. (B,C) A synapse organizer is produced in the presynaptic cell by the activity of an independent transcription factor (model 2) or a terminal selector (model 3). See Introduction for a detailed description.

In the case of GABAergic neurons, NT identity is defined by the co-expression of highly conserved proteins, including (1) the enzyme glutamic acid decarboxylase (GAD) (Sgado et al., 2011), which synthesizes GABA from its precursor, (2) the vesicular GABA transporter (VGAT), which packages GABA into synaptic vesicles, and (3) the GABA re-uptake transporter (GAT) (Fig. 1) (Gendrel et al., 2016). Importantly, reduced expression of these GABA identity determinants, as well as impaired GABA transmission, lead to a variety of neuropsychiatric diseases, including schizophrenia, autism, epilepsy and anxiety (Zhou and Bessereau, 2019; Bolneo et al., 2022).

Despite GABA being the most abundant inhibitory NT in both invertebrate and vertebrate nervous systems, it is poorly understood how the expression of GABA identity genes is controlled over time, from development through to adulthood, to ensure GABA neurotransmission. To date, a handful of studies in *C. elegans* and mice have identified terminal selectors in various GABAergic neuron types. Examples include homeodomain proteins (e.g. UNC-30/PITX) (Cinar et al., 2005; Eastman et al., 1999; Jin et al., 1994; Westmoreland et al., 2001), nuclear hormone receptors (e.g. NHR-67/NR2E1) (Gendrel et al., 2016) and GATA-type (GATA2/3) transcription factors, each necessary for expression of GABA identity genes during development (Kala et al., 2009; Lahti et al., 2016; Yang et al., 2010). However, whether any of these factors is required for maintaining GABA identity gene expression during post-embryonic life is unknown (Achim et al., 2014).

During neuronal development, GABA receptor (GABAR) clustering is fundamental for postsynaptic differentiation, a process primarily driven by synapse-organizing molecules that can be either secreted or bound to the cell membrane (Zhou and Bessereau, 2019). In mice, the cell adhesion molecule neuroligin 2, the scaffold protein gephyrin and the transmembrane protein β-dystroglycan act as synapse organizers to control GABAR clustering (Briatore et al., 2020; Essrich et al., 1998; Soykan et al., 2014). In *C. elegans*, the secreted molecule MADD-4 (Muscle Arm Development Defect-4) (ortholog of human punctin/ADAMTSL), acts as an anterograde synapse organizer at neuromuscular synapses (Pinan-Lucarre et al., 2014). Specifically, the short MADD-4 isoform (MADD-4B) activates UNC-40/DCC (deleted in colorectal cancer) signaling, recruiting an intracellular postsynaptic scaffold composed of FRM-3, a FERM domain protein, and LIN-2/CASK (Tu et al., 2015; Zhou et al., 2020). Moreover, MADD-4B controls GABAR positioning at synapses by recruiting the sole *C. elegans* neuroligin homolog, NLG-1, which binds to LIN-2 (Maro et al., 2015; Platsaki et al., 2020; Zhou et al., 2020; Tu et al., 2015). By contrast, the long *madd-4* isoform, MADD-4L, promotes the clustering of levamisole-sensitive ACh receptors (L-AChRs) on muscle cells through the formation of an extracellular scaffold (Gally et al., 2004; Gendrel et al., 2009; Pinan-Lucarre et al., 2014; Rapti et al., 2011).

In vertebrates, there are two *madd-4* orthologs: *Adamtsl1* (previously known as punctin-1) and *Adamtsl3* (previously known as punctin-2) (Hall et al., 2003; Hirohata et al., 2002). A recent study identified Adamtsl3 as an extracellular synapse organizer in the rodent hippocampus, where it supports glutamatergic and GABAergic synapse formation *in vivo* (Cramer et al., 2023). In the adult mouse brain, Adamtsl3 signals via DCC at GABAergic synapses and facilitates synapse maintenance, synaptic plasticity, and memory. In humans, *ADAMTSL3* is widely expressed in the brain, and has been identified as a candidate gene for schizophrenia (Dow et al., 2011). Despite their well-established roles in GABAR clustering, the transcriptional mechanisms that control expression of synapse organizers remain poorly understood.

GABA neurotransmission relies on (1) the ability of the presynaptic neuron to continuously express GABA identity genes (e.g. GAD, VGAT, GAT) and (2) the ability of the postsynaptic neurons to cluster GABARs appropriately (Gravielle, 2021; Mizumoto et al., 2023). Whether these two processes, which occur in two synaptically connected cells, are coordinated remains poorly understood. In principle, at least three non-mutually exclusive models can be envisioned for the transcriptional control of a GABA synapse organizer (Fig. 1). GABAR clustering at the post-synaptic (target) cell could be achieved via the activity of a synaptic organizer (membrane-bound or secreted) produced in the post-synaptic cell. For example, gephyrin, a synapse organizer produced in the target cell, is essential for GABAR clustering (Fig. 1A, model 1). Alternatively, GABAR clustering in the post-synaptic cell may rely on secreted synaptic organizers, such as MADD-4/punctin, produced in the presynaptic GABAergic neuron (Fig. 1B,C, models 2 and 3). In that case, transcription of the synapse organizer gene may or may not require the activity of the terminal selector of the presynaptic neuron (model 2 versus 3). Our previous work in *C. elegans* provided support for model 3 in cholinergic neuromuscular synapses (Kratsios et al., 2015); the terminal selector UNC-3 (Collier, Ebf) is not only required for AChR clustering in the postsynaptic neuron, but also controls NT identity genes in the presynaptic cell. However, whether this principle of transcriptional coordination extends beyond cholinergic motor neurons (MNs) was unclear.

*C. elegans* has been a prime model to dissect molecular mechanisms underlying NT identity and synapse formation (Hobert, 2016; Mizumoto et al., 2023). Here, we show that the *C. elegans* terminal selector of GABAergic nerve cord MNs, UNC-30, is required for clustering of type A GABARs, a major type of inhibitory NT receptors (Ghit et al., 2021; Goetz et al., 2007). We find that UNC-30 acts directly to activate transcription of the synapse organizer *madd-4B*. Hence, the terminal selector UNC-30 coordinates GABAR clustering on postsynaptic muscle cells (via control of *madd-4B*) with acquisition of GABAergic identity in presynaptic MNs (Fig. 1, model 3). Further, we find that UNC-30 acts directly to maintain the expression of *madd-4B* and NT identity genes (e.g. *unc-25*, *unc-47*) in late larval and adult stages. Intriguingly, UNC-30 also represses transcription of the long *madd-4* isoform (*madd-4L)*, which is normally required for AChR clustering in postsynaptic muscle cells (Pinan-Lucarre et al., 2014). Hence, our work in GABA MNs highlights that NT receptor clustering, a central event of postsynaptic differentiation, is transcriptionally coordinated with acquisition and maintenance of NT identity, significantly extending previous observations made in *C. elegans* cholinergic MNs to other neuron types (Kratsios et al., 2015). Last, we uncovered additional target genes that are either positively or negatively regulated by UNC-30, indicating both activator and repressor functions. Such mechanistic insights may help us understand the molecular mechanisms underlying human genetic disorders caused by *PITX* gene mutations, such as Axenfeld–Rieger syndrome (Muzyka et al., 2023; Tumer and Bach-Holm, 2009; Tran and Kioussi, 2021).

## RESULTS

### The experimental system: GABAergic neuromuscular synapses in *C. elegans*

**Fig. 2.**
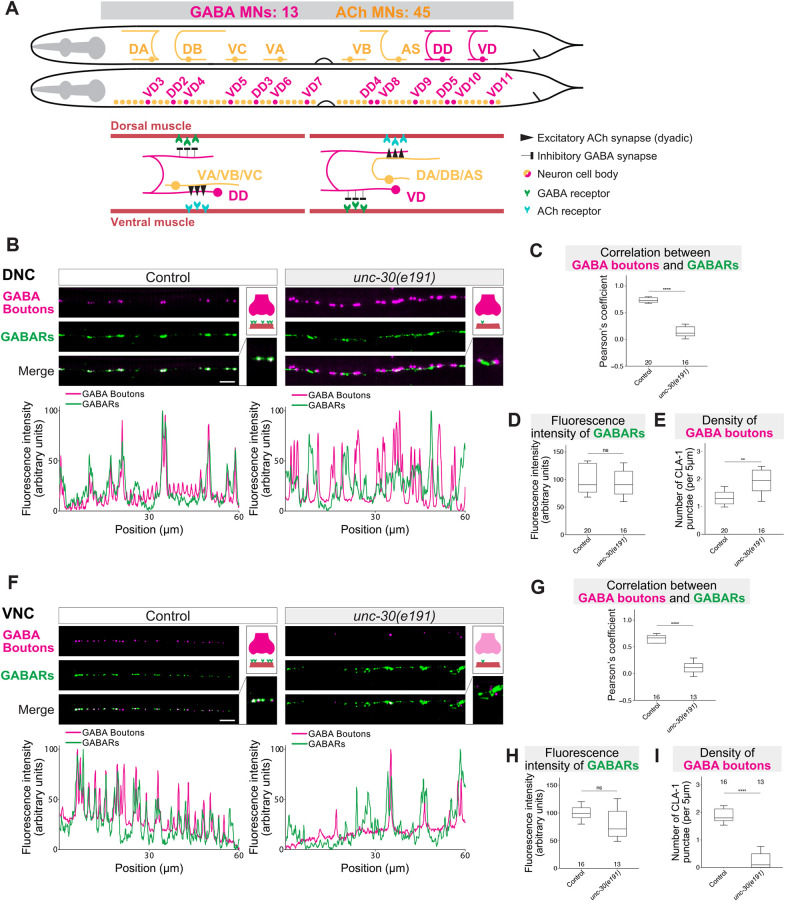
***unc-30* is required for proper clustering of GABA_A_ receptors at *C. elegans* neuromuscular synapses.** (A) Cholinergic (DA, DB, VC, VA, VB, AS) and GABAergic (DD, VD) MNs in the *C. elegans* VNC. (B,F) Fluorescence micrographs of GABAergic presynaptic boutons (*bab462 [cla-1::GFP11×7]; krSi342 [unc-30prom::gfp1-10]*; magenta) and GABA_A_Rs (*kr296 [unc-49::rfp]*; green) in control and *unc-30(e191)* animals (B, DNC; F, VNC). Fluorescence intensity profiles are shown below. Insets on the right show higher-magnification images and schematics representing GABAergic presynaptic boutons (magenta), body wall muscle cells (pink), and GABA_A_Rs (green). Scale bars: 5 µm. (C,G) Pearson's correlation coefficient between GABA_A_Rs (UNC-49-RFP) and GABAergic boutons (CLA-1-GFP) as shown in B and F. Mann–Whitney test. ****P<0.0001. (D,H) Fluorescence intensity of GABA_A_Rs (UNC-49-RFP) as shown in B and F. Student's *t*-test (D) and Mann–Whitney test (H). (E,I) Density of GABAergic boutons as shown in B and F. Mann–Whitney test. **P<0.01, ****P<0.0001. Box and whisker plots show median, and lower and upper quartiles; whiskers represent s.d. Number of worms analyzed is indicated on box plots. ns, not significant.

*C. elegans* locomotion relies on both cholinergic and GABAergic MNs, the cell bodies of which intermingle along the ventral nerve cord (VNC) (Fig. 2A). Based on anatomical criteria, cholinergic and GABAergic MNs are respectively divided into six (VA, VB, DA, DB, AS, VC) and two (DD, VD) classes, which form *en passant* synapses along the ventral and dorsal nerve cords (Fig. 2A) (Mizumoto et al., 2023; Von Stetina et al., 2006). The coordinated activity of excitatory cholinergic and inhibitory GABAergic MNs generates sinusoidal locomotion, with each muscle cell receiving dual innervation from cholinergic and GABAergic MNs. Along the dorsal nerve cord (DNC) of adult animals, three cholinergic MN classes (DA, DB and AS) form dyadic synapses, providing excitatory input not only to dorsal muscles but also to VD GABAergic neurons, which in turn innervate and inhibit ventral muscles (Fig. 2A) (White et al., 1986). Along the VNC, another three cholinergic MN classes (VA, VB and VC) also form dyadic synapses with ventral muscles and DD GABAergic neurons, which innervate and inhibit dorsal muscles (Fig. 2A). Because each muscle cell receives both excitatory (ACh) and inhibitory (GABA) inputs, the *C. elegans* neuromuscular system represents a powerful model in which to study how different NT receptors precisely cluster in front of their corresponding neurotransmitter release sites (Mizumoto et al., 2023).

### *unc-30* controls GABA_A_ receptor clustering at inhibitory neuromuscular synapses

Within the *C. elegans* VNC, the transcription factor UNC-30 is specifically expressed in GABAergic (DD, VD) MNs (Jin et al., 1994), where it controls expression of GABA identity genes (Fig. 1) (McIntire et al., 1993; Cinar et al., 2005; Eastman et al., 1999; Jin et al., 1994; Westmoreland et al., 2001). Recent studies also implicated UNC-30 in synaptic remodeling, as it prevents premature synapse rewiring of DD cells and aberrant synapse rewiring of VD cells (He et al., 2015; Howell et al., 2015). However, whether UNC-30 is necessary for the postsynaptic differentiation of target muscle cells remained unknown.

We therefore investigated whether genetic loss of *unc-30* affects GABAR clustering in *C. elegans* muscle cells innervated by GABAergic MNs. We used an endogenous *RFP* reporter for *unc-49* (UNC-49::RFP), which encodes a type-A GABAR (GABA_A_R) expressed in both ventral and dorsal body wall muscles (Fig. 2B) (Zhou et al., 2020). To visualize the presynaptic boutons of GABAergic MNs (DD, VD), we employed native and tissue-specific fluorescence (NATF) (He et al., 2019), resulting in GFP labeling of endogenous CLA-1 (Clarinet), an active zone protein (Xuan et al., 2017). Using these tools (Fig. 2), as well as an additional presynaptic marker (RAB-3::GFP) (Fig. S1A,B), we visualized in young adult (day 1) animals the juxtaposition of GABAergic presynaptic boutons and GABA_A_R clusters in body wall muscles along the DNC (DD neuromuscular synapses) (Fig. 2B-E, Fig. S1A,B) and VNC (VD neuromuscular synapses) (Fig. 2F-I).

In homozygous adult (day 1) animals carrying a strong loss-of-function (LOF) *unc-30* allele, *e191* (Jin et al., 1994; Brenner, 1974), we found that GABA_A_Rs are present on dorsal muscle (DNC), but no longer cluster opposite presynaptic GABA boutons of DD neurons (Fig. 2B,C, Fig. S1A,B). The levels of UNC-49::RFP expression in dorsal muscle cells were not affected in *unc-30(e191)* animals (Fig. 2D), suggesting that the GABA_A_R clustering phenotype is not due to decreased UNC-49 expression. Similarly, we observed GABA_A_R clustering defects and no effect on UNC-49 expression along the VNC of *unc-30(e191)* animals (Fig. 2F-H), indicating that VD neuromuscular synapses are also affected in the absence of *unc-30*. Although the correlation of GABA boutons and GABA_A_Rs was lower at both the DNC and the VNC of *unc-30(e191)* mutants (Fig. 2C-G), the density of GABA boutons was higher at the DNC and lower at the VNC, consistent with previously reported wiring defects in *unc-30(e191)* animals (Howell et al., 2015). Altogether, we conclude that *unc-30* is required for GABA_A_R clustering at neuromuscular synapses of GABAergic (DD, VD) neurons.

### GABA_A_ receptors in *unc-30* mutants are juxtaposed to cholinergic boutons at neuromuscular synapses

**Fig. 3.**
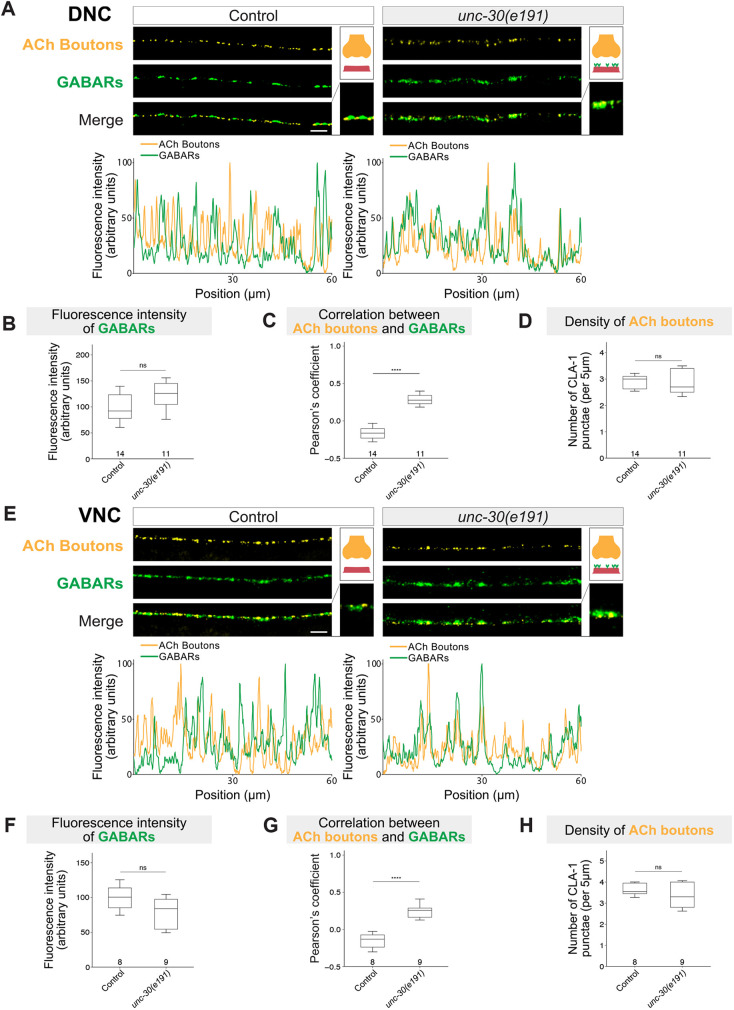
**GABA_A_ receptors cluster at excitatory neuromuscular synapses in *unc-30* mutants.** (A,E) Fluorescence micrographs of cholinergic presynaptic boutons (*krSi144[unc-17prom::cla-1::bfp*; yellow*]*) and GABA_A_Rs (*kr296 [unc-49::rfp]*; green) in control and *unc-30(e191)* animals (A, DNC; E, VNC). Fluorescence intensity profiles are shown below. Insets on the right show higher-magnification images and schematics representing cholinergic presynaptic boutons (yellow), body wall muscle cells (pink), and GABA_A_Rs (green). Scale bars: 5 µm. (B,F) Fluorescence intensity of GABA_A_Rs (UNC-49-RFP) as shown in A and E. Mann–Whitney test (B) and Student's *t*-test (F). (C,G) Pearson's correlation coefficient between GABA_A_Rs (UNC-49-RFP) and cholinergic boutons (CLA-1-BFP) as shown in A and E. Mann–Whitney test. ****P<0.0001. (D,H) Density of cholinergic boutons (CLA-1-BFP) as shown in A and E. Unpaired, two-tailed Student's *t*-test. ns, not significant.

Because in control animals GABAergic (DD) and cholinergic (DA, DB, AS) neurons form *en passant* neuromuscular synapses with dorsal muscles at the DNC (Fig. 2A), we considered the possibility that, in *unc-30(e191)* mutants, GABA_A_Rs not only fail to cluster properly across GABA boutons (Fig. 2C,G), but are also inappropriately juxtaposed to presynaptic boutons of cholinergic (DA, DB, AS) MNs. To test the latter, we genetically labeled cholinergic presynaptic boutons with CLA-1::BFP and GABA_A_Rs with UNC-49::RFP, and found that GABA_A_Rs incorrectly localize opposite to cholinergic presynaptic boutons in *unc-30(e191)* mutants (Fig. 3A,C). Double immunofluorescence staining against UNC-49 and UNC-17 (VAChT/SLC18A3), another marker of cholinergic presynaptic boutons, yielded similar results at the DNC (Fig. S1C). Importantly, we also observed that GABA_A_Rs incorrectly localize opposite to cholinergic boutons at the VNC (Fig. 3E-G), indicating that the GABA_A_R clustering defects are present both in VD and DD neuromuscular synapses. Notably, loss of *unc-30* did not affect the density of cholinergic boutons (Fig. 3D,H) or UNC-49 expression (Fig. 3B,F) at the DNC and VNC.

We next wondered whether the aberrant GABA_A_R clustering opposite to cholinergic boutons affects structural features of the cholinergic neuromuscular synapse. However, we found that the L-AChR UNC-29 normally localizes opposite to cholinergic boutons in *unc-30(e191)* mutants (Fig. S1D,F). Additional synaptic features, such as UNC-29::RFP expression on the muscle and density of presynaptic ACh boutons, were also not affected (Fig. S1E,G).

Altogether, *unc-30* is necessary for the correct positioning of GABA_A_Rs at neuromuscular synapses of the DNC and VNC. Because *unc-30* is present in GABAergic MNs but not expressed in body wall muscles or muscle progenitor cells (Figs S2, S3) (Jin et al., 1994), it is likely that *unc-30* controls GABA_A_R clustering in an indirect (non-cell-autonomous) manner.

### The short isoform of *madd-4* controls GABA_A_R clustering at neuromuscular synapses in a non-cell-autonomous manner

**Fig. 4.**
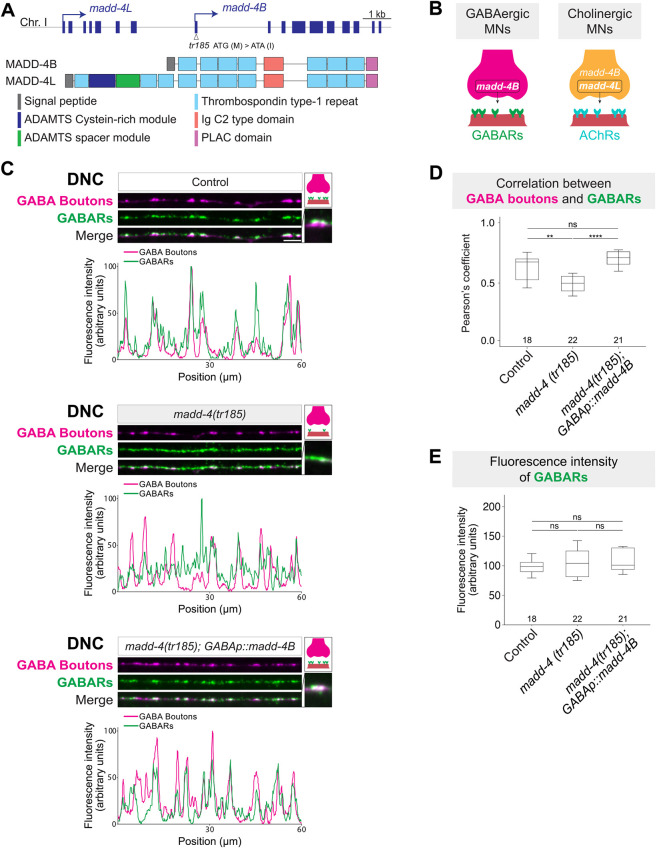
***madd-4B* is required for specific positioning of GABA_A_Rs at inhibitory neuromuscular synapses.** (A) Schematic of the *madd-4* locus and MADD-4B/L protein domains. *madd-4(tr185)* is a mutation in exon 1 of *madd-4B* (empty arrowhead), converting ATG (Met) to ATA (Ile). (B) Schematic of *madd-4B*/*L* expression and function in MNs. (C) Fluorescence micrographs of GABAergic presynaptic boutons (*krIs67[unc-47prom::snb-1::bfp]*; magenta) and GABA_A_Rs (*kr296 [unc-49::rfp]*; green) along the DNC in control and *madd-4(tr185)* animals, as well as in a rescue strain expressing *madd-4B* in GABAergic MNs (*krSi92 [unc-47prom::T7::madd-4B::gfp]*). Fluorescence intensity profiles are shown below. Insets on the right show higher-magnification images and schematics representing GABAergic presynaptic boutons (magenta), body wall muscle cells (pink), and GABA_A_Rs (green). Scale bars: 5 µm. (D) Pearson's correlation coefficient between UNC-49-RFP and SNB-1-BFP from the data shown in C. Kruskal–Wallis followed by Dunn's post-test. **P<0.01, ****P<0.0001. (E) Fluorescence intensity of UNC-49-RFP from the data shown in C. Kruskal–Wallis followed by Dunn's post-test. ns, not significant.

We previously demonstrated that *madd-4*, a secreted synapse organizer, is required for GABA_A_R and AChR clustering at *C. elegans* neuromuscular synapses (Pinan-Lucarre et al., 2014). The *madd-4* locus generates two isoforms through alternative promoter usage (Fig. 4A) (Kratsios et al., 2015; Seetharaman et al., 2011). The long isoform (*madd-4L*) is produced by cholinergic MNs and required for L-AChR clustering at neuromuscular synapses (Fig. 4B) (Kratsios et al., 2015; Pinan-Lucarre et al., 2014). The short isoform (*madd-4B*) is required for GABA_A_R clustering (Fig. 4B) (Pinan-Lucarre et al., 2014). Because *madd-4B* is produced by both GABAergic and cholinergic MNs (Kratsios et al., 2015; Pinan-Lucarre et al., 2014; Tu et al., 2015), it remained unclear whether *madd-4B* from GABAergic and/or cholinergic MNs is required for GABA_A_R clustering at neuromuscular synapses.

To test this, we first analyzed animals specifically lacking *madd-4B* gene activity using the *madd-4(tr185)* allele (Fig. 4A). Confirming their previously reported synaptic phenotype (Tu et al., 2015; Zhou et al., 2020), we found that UNC-49::RFP fluorescence signal on the dorsal muscle of *madd-4B(tr185)* animals was no longer restricted to sites opposite GABA (DD) boutons (Fig. 4C). We indeed found that the correlation of GABA_A_R localization and GABA boutons is lower in *madd-4(tr185)* animals (Fig. 4D). Importantly, GABA_A_Rs (visualized with UNC-49::RFP) were detected both at and between GABAergic presynaptic boutons along the DNC of *madd-4B(tr185)* animals (Fig. 4C). Because both GABAergic and cholinergic neuromuscular synapses are located *en passant*, the continuous distribution of UNC-49::RFP along the DNC suggests that GABA_A_R clusters face both GABAergic (DD) and cholinergic (DA, DB, AS) presynaptic boutons (Fig. 2A). This is likely due to remaining MADD-4L expression in cholinergic MNs of *madd-4B(tr185)* mutants, leading to ectopic GABA_A_R trapping at cholinergic neuromuscular synapses (Pinan-Lucarre et al., 2014). The aberrant clustering of GABA_A_Rs was not accompanied by an increase in UNC-49 expression levels on the muscle (Fig. 4E). Importantly, expression of *madd-4B* specifically in GABAergic MNs led to a complete rescue of the GABA_A_R clustering defects at postsynaptic muscle cells, consolidating a non-cell-autonomous role for *madd-4B*.

### UNC-30 controls *madd-4B* transcription in GABAergic MNs

**Fig. 5.**
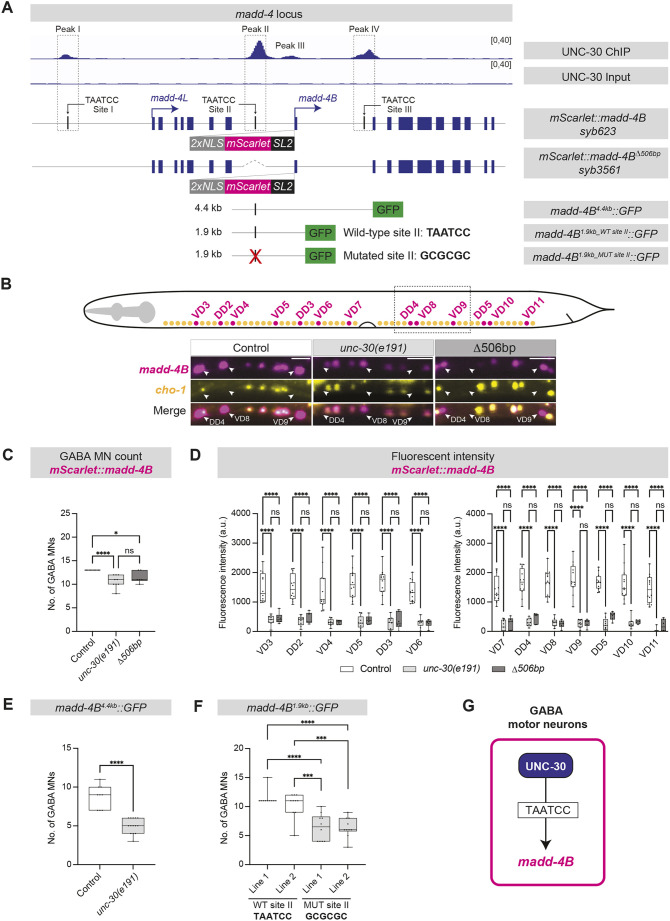
**UNC-30 directly activates *madd-4B* in GABAergic MNs.** (A) UNC-30 ChIP-Seq and Input (negative control) on the *madd-4* locus. Four UNC-30 binding peaks (peaks I, II, III, IV) and three UNC-30 binding sites (TAATCC sites I, II, III) are shown. (B) GABAergic (magenta) and cholinergic (yellow) MNs. Dashed box in schematic depicts the imaged area. Fluorescence micrographs of *madd-4B* (*syb623[2xNLS::mScarlet::SL2::madd-4B]*) and a cholinergic MN reporter (*otIs354[cho-1(fosmid)::SL2::YFP::H2B]*) in control and *unc-30(e191)* animals, and *syb3561[2xNLS::mScarlet::SL2:: madd-4B^Δ506bp^]* animals. GABA MNs: *mScarlet+;YFP–*; cholinergic MNs: *mScarlet+;YFP+*. Images of day 1 adults. White arrowheads indicate GABAergic MNs. Scale bars: 10 µm. (C) Quantification of GABAergic MN numbers in the genotypes shown in B. Unpaired *t*-test with Welch's correction. *P<0.05, ****P<0.0001. Control: *n*=10; *unc-30(e191)*: *n*=10; Δ506 bp mutant: *n*=8. (D) Quantification of *madd-4B* (*2xNLS::mScarlet::SL2::madd-4B*) fluorescence intensity in individual GABAergic MNs. Two-way ANOVA followed by Sidak's multiple comparison test. ****P<0.0001. Control: *n*=10; *unc-30(e191)*: *n*=10; Δ506 bp mutant: *n*=8. (E) Quantification of GABAergic MNs expressing *madd-4B* (*otEx5601[madd-4B^4.4kb^::GFP]*) in control and *unc-30(e191)* animals. Unpaired *t*-test with Welch's correction. ****P<0.0001. Control: *n*=9; *unc-30(e191)*: *n*=14. (F) Quantification of GABAergic MNs expressing *otEx4948-9[madd-4B^1.9kb_TAATCC^::GFP]* and *kasEx315-6[madd-4B^1.9kb_GCGCGC^::GFP]s.* One-way ANOVA followed by Sidak's multiple comparison test. ***P<0.001, ****P<0.0001. Wild-type (WT) line 1: *n*=10; wild-type line 2: *n*=10; *madd-4B^1.9kb_GCGCGC^* line 1: *n*=10; *madd-4B^1.9kb_GCGCGC^* line 2: *n*=10. (G) Schematic showing that UNC-30 directly activates *madd-4B*. Box and whisker plots in C-F show median, and lower and upper quartiles; whiskers represent minimum and maximum. Black circles depict individual values. ns, not significant (*P*>0.05).

Because both *unc-30* and *madd-4B* mutants display defects in GABA_A_R localization (Figs 2-4), we hypothesized that the transcription factor UNC-30 regulates *madd-4B* in GABAergic MNs. To test this, we employed CRISPR/Cas9 genome editing and generated an endogenous fluorescent reporter of *madd-4B* transcription. In agreement with transgenic *madd-4B* reporter expression (Kratsios et al., 2015), this endogenous *2xNLS::mScarlet::SL2::madd-4B* transcriptional reporter (*mScarlet::madd-4B* hereafter) was expressed in both cholinergic and GABAergic MNs, although higher levels were observed in GABAergic MNs (Fig. 5A,B). To test the effect of *unc-30* gene loss in *madd-4B* expression specifically in GABAergic MNs, we crossed a nuclear marker for cholinergic MNs (*cho-1::SL2::YFP::H2B*) to the *mScarlet::madd-4B* reporter in the context of control and *unc-30(e191)* animals (Fig. 5B). We observed a significant decrease in the number of GABAergic cells (defined by the absence of *cho-1::SL2::YFP::H2B* signal) expressing *mScarlet::madd-4B* in *unc-30(e191)* mutants at the fourth larval (L4) stage (Fig. 5B,C), i.e. all 13 GABAergic neurons (DD2-DD5, VD3-VD11) of the VNC expressed *mScarlet::madd-4B* in control animals, but only around ten neurons in *unc-30(−)* mutants (Fig. 5A,C). Importantly, the remaining *mScarlet::madd-4B* expression in these ten GABAergic neurons was also decreased, as revealed by quantification of *mScarlet::madd-4B* fluorescence intensity with single-cell resolution (e.g. VD3, DD2, VD4, VD5, DD3, VD6) (Fig. 5D). The remaining *madd-4B* expression suggests that additional, yet-to-be-identified factors cooperate with UNC-30 to activate *madd-4B* expression in these cells. In our analysis, we excluded six GABAergic MNs (DD1, DD6, VD1, VD2, VD13) because their location (outside the VNC) makes their identification less straightforward.

The single-cell resolution of our analysis indicates that *unc-30* controls *madd-4B* transcription in both DD (e.g. DD2, DD3) and VD (e.g. VD3, VD4, VD5) neurons (Fig. 5D). To corroborate this, we quantified *mScarlet::madd-4B* expression at larval stage 1 (L1), a developmental stage at which only DD (not VD) neurons are present in the *C. elegans* nerve cord (Fig. S4A). Again, we found a significant decrease in *madd-4B* expression in DD neurons of *unc-30(e191)* mutants (Fig. S4B). In agreement with our endogenous transcriptional reporter (*mScarlet::madd-4B*), expression of a transgenic translational *madd-4B* reporter is also affected in *unc-30* animals at L1 (Chen et al., 2021). Altogether, we conclude that *unc-30* controls endogenous *madd-4B* transcription in GABAergic MNs, and this effect is observed both at early (L1) and late (L4) larval stages.

### UNC-30 directly activates *madd-4B* transcription in GABAergic MNs

Because *madd-4B* expression is reduced in GABA MNs of *unc-30(e191)* animals (Fig. 5B-D), we investigated whether *madd-4B* is a direct target of UNC-30. Leveraging an available dataset of chromatin immunoprecipitation followed by sequencing (ChIP-Seq) (Yu et al., 2017), we identified UNC-30 binding at four genomic regions (peaks I-IV): peak I is upstream of *madd-4L*, whereas peaks II-IV surround the first exon of *madd-4B* (Fig. 5A). Within peaks I, II and IV, we identified a canonical UNC-30 binding site (TAATCC) (Cinar et al., 2005; Eastman et al., 1999). To test whether UNC-30 binding upstream of *madd-4B* is required for *madd-4B* expression, we employed CRISPR/Cas9 genome editing to delete a 506 bp-long region that spans peak II (Δ506 bp; Fig. 5A). This manipulation was conducted in animals carrying the endogenous *mScarlet::madd-4B* reporter. Similar to *unc-30(e191)* mutants, we observed a decrease in the number of GABAergic MNs expressing *mScarlet* in L4 stage animals homozygous for the 506 bp deletion (Fig. 5B,C), and in the levels of *mScarlet* expression in individual GABAergic MNs (Fig. 5D).

ChIP-Seq data and our analysis of *mScarlet::madd-4B*^Δ*506bp*^ animals strongly indicate that UNC-30 acts directly to activate *madd-4B* transcription. To further test this possibility, we examined transgenic animals carrying different transcriptional reporters of *madd-4B* (Fig. 5A). First, we found that reporters containing DNA sequences either 4.4 kb (*madd-4B^4.4kb^::GFP*) or 1.9 kb (*madd-4B^1.9kb^::GFP*) upstream of *madd-4B* (both containing peak II) drive *GFP* expression in GABA MNs (Fig. 5A,E,F), consistent with the endogenous *madd-4B::mScarlet* reporter (Fig. 5C). Second, *madd-4B^4.4kb^::GFP* reporter expression depends on *unc-30*, evidenced by a reduction in the number of GABA MNs expressing *GFP* in *unc-30(e191)* mutants (Fig. 5E). Third, we found that mutation of the UNC-30 binding site II (wild type: TAATCC; mutated: GCGCGC) results in a significant decrease in the number of GABA MNs expressing *madd-4B^1.9kb^::GFP* (Fig. 5F). Altogether, we conclude that UNC-30 acts directly to activate *madd-4B* transcription in GABA MNs (Fig. 5G).

### UNC-30 represses *madd-4L* transcription in GABAergic MNs

**Fig. 6.**
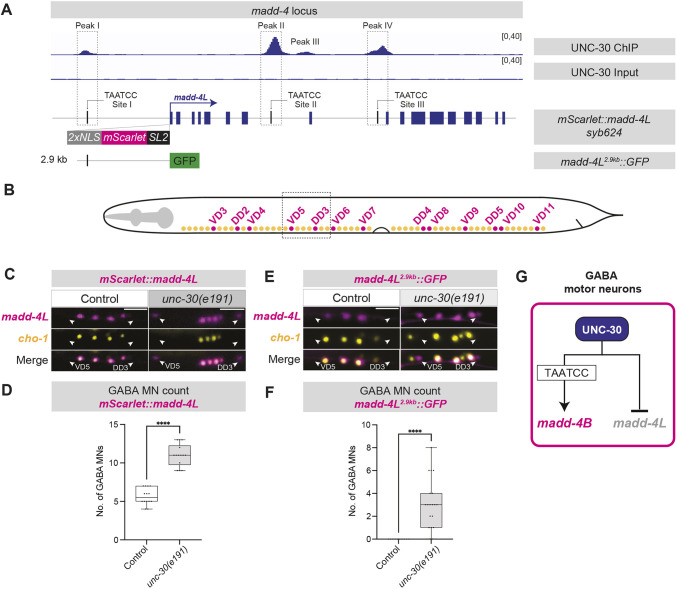
**UNC-30 represses *madd-4L* in GABAergic motor neurons.** (A) UNC-30 ChIP-Seq and Input (negative control) tracks on the *madd-4* locus. Depicted below: (1) endogenous (*syb624[2xNLS::mScarlet::SL2::madd-4L]*) and 2) transgenic (*sEx14990 [madd-4L(2.9 kb prom)::GFP]*) *madd-4L* reporters. (B) GABAergic (magenta) and cholinergic (yellow) MNs in *C. elegans*. Dashed box depicts the imaged area shown in C and E. (C) Fluorescence micrographs of *madd-4L* (*syb624 [2xNLS::mScarlet::SL2::madd-4L]*) and a cholinergic motor neuron reporter (*otIs354[cho-1(fosmid)::SL2::YFP::H2B]*) in control and *unc-30(e191)* animals. GABAergic MNs: *mScarlet+*;*YFP–*, cholinergic MNs: *mScarlet+*;*YFP+*. Images of day 1 adults. White arrowheads indicate GABAergic MNs. Scale bars: 10 µm. (D) Quantification of GABAergic MNs expressing *madd-4L(syb624 [2xNLS::mScarlet::SL2::madd-4L])* as shown in C. Unpaired *t*-test with Welch's correction. ****P<0.0001. Wild type: *n*=13; *unc-30(e191)*: *n*=13. (E) Fluorescence micrographs of *madd-4L* (*sEx14990[madd-4L(2.9 kb prom)::GFP]*) and a cholinergic MN reporter (*otIs544 [cho-1(fosmid)::SL2::mCherry::H2B]*) in control and *unc-30(e191)* animals. GABAergic MNs: *GFP+*;*mCherry* –; cholinergic MNs: *GFP+*;*mCherry+*. Images of day 1 adults. White arrowheads indicate GABAergic MNs. Scale bar: 10 µm. (F) Quantification of the number of GABAergic MNs expressing *madd-4L* (*sEx14990 [madd-4L(2.9 kb prom)::GFP]*) as shown in E. Unpaired *t*-test with Welch's correction. ****P<0.0001. Wild type: *n*=13; *unc-30(e191)*: *n*=13. (G) Schematic showing the dual role of UNC-30 in controlling *madd-4* isoforms.

The ChIP-Seq data also showed UNC-30 binding (peak I) upstream of exon 1 of *madd-4L* (Fig. 6A). Because *madd-4L* is known to be specifically expressed in cholinergic MNs (Kratsios et al., 2015; Pinan-Lucarre et al., 2014), we hypothesized that UNC-30 binds upstream of *madd-4L* to repress its transcription in GABA MNs. Supporting this notion, transgenic GFP animals carrying a 2.9 kb sequence upstream of *madd-4L* showed increased expression in GABA MNs of *unc-30(e191)* mutants (Fig. 6E,F). Next, we employed CRISPR/Cas9 genome editing and generated an endogenous *mScarlet* reporter for *madd-4L* (*syb624[2xNLS::mScarlet::SL2::madd-4L]*), referred to hereafter as *mScarlet::madd-4L* (Fig. 6A). We observed ectopic expression of *mScarlet::madd-4L* in GABA MNs of *unc-30(e191)* mutant animals both at L1 (Fig. S5A,B) and at L4 (Fig. 6B-D). We found that up to 13 GABA MNs of the VNC ectopically express *mScarlet::madd-4L* in *unc-30(e191)* mutants (Fig. 6B-D). However, mutating the endogenous UNC-30 binding sequence (WT site I: TAATCC site; MUT site I: GCGCGC) within peak I had no effect on the number of GABA MNs expressing *mScarlet::madd-4L* (Fig. S5C,D). We conclude that, in GABA MNs, UNC-30 controls two isoforms of the same synapse organizer in opposite ways: it directly activates *madd-4B* and indirectly represses *madd-4L* (Fig. 6G).

### UNC-30 is continuously required to maintain *madd-4B* expression in GABAergic MNs

**Fig. 7.**
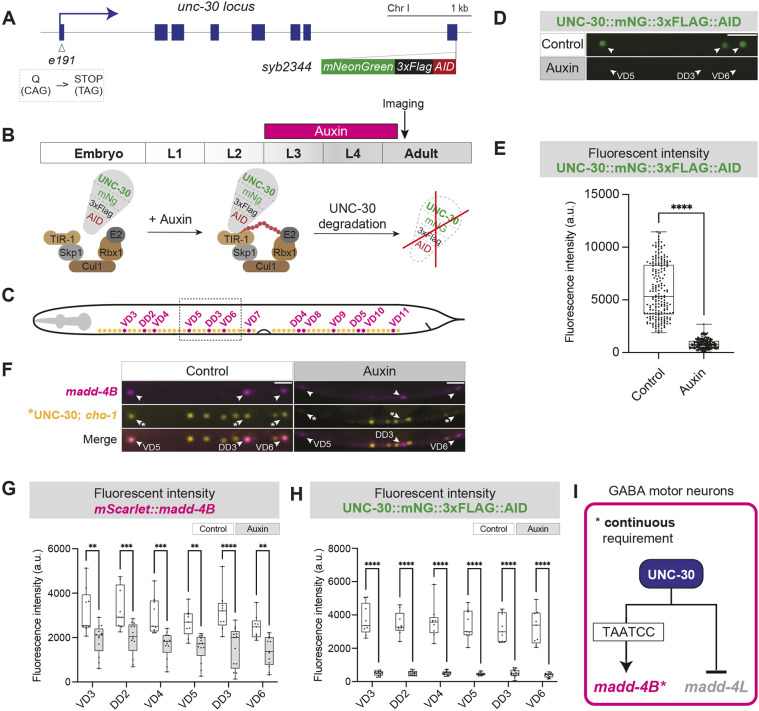
**UNC-30 is required to maintain *madd-4B* expression in GABA MNs.** (A) Schematic of the *unc-30* locus, and *e191* and *syb2344* (*unc-30::mNG::3xFLAG::AID*) alleles. *e191* converts CAG (Q) to TAG (STOP) at codon 16. (B) Auxin treatment timeline. Day 1 adults were imaged. The E3 ligase complex: Skp1, Cul1, Rbx1, E2. (C) Schematic of *C. elegans* with dashed box depicting the imaged area shown in D and F. (D) Fluorescence micrographs of UNC-30 (*syb2344 [UNC-30::mNG::3xFLAG::AID]*) in control (EtOH) and auxin-treated animals expressing TIR1 pan-somatically (*ieSi57 [Peft-3::TIR1::mRuby]*). White arrowheads indicate GABAergic MNs. (E) Quantification of UNC-30 (*syb2344 [UNC-30::mNG::3xFLAG::AID]*) fluorescence intensity in GABAergic MNs. Unpaired *t*-test with Welch's correction. ****P<0.0001. Control: *n*=195 MNs; auxin-treated: *n*=195 MNs. (F) Fluorescence micrographs of *madd-4B* (*syb623[2xNLS::mScarlet::SL2::madd-4B]*), UNC-30 (*syb2344[UNC-30::mNG::3xFLAG::AID]*) and a cholinergic MN reporter (*otIs354[cho-1(fosmid)::SL2::YFP::H2B]*) in control (EtOH treated) and auxin-treated animals. White arrowheads indicate GABAergic MNs. Asterisks indicate GABA MNs expressing UNC-30::mNG::3xFLAG::AID. Scale bars: 10 µm. (G,H) Quantification of *madd-4B* (*2xNLS::mScarlet::SL2::madd-4B*) (G) or UNC-30 (*syb2344 [UNC-30::mNG::3xFLAG::AID]*) (H) fluorescence intensity in GABAergic MNs, as shown in F. Two-way ANOVA followed by Sidak's multiple comparison test. **P<0.002, ***P<0.0002, ****P<0.0001. Control: *n*=9; auxin treated: *n*=13. (I) Schematic showing that UNC-30 is required to maintain *madd-4B*.

The continuous expression of both *unc-30* and *madd-4B* in GABAergic MNs, at larval stages and throughout adulthood, raises the question of whether UNC-30 is required continuously to activate *madd-4B* expression. We therefore generated an inducible *unc-30* allele, leveraging the auxin-inducible degradation (AID) system (Towbin et al., 2012; Ashley et al., 2021; Zhang et al., 2015). Using CRISPR/Cas9, we introduced the *mNG::3xFLAG::AID* cassette before the *unc-30* STOP codon (Fig. 7A). The resulting *unc-30::mNG::3xFLAG::AID* allele *(syb2344)* serves as an endogenous fluorescent (mNG, mNeonGreen) reporter of UNC-30, which can be degraded upon auxin treatment owing to the presence of the AID degron (Fig. 7B). We generated double-homozygous animals for *unc-30::mNG::3xFLAG::AID* and *ieSi57 (Peft-3::TIR1::mRuby)*, the latter providing pan-somatic expression of *TIR1* – an F-box protein that binds to AID in the presence of auxin, leading to proteasomal degradation of UNC-30::mNG::3xFLAG::AID. As proof of principle, we first assessed UNC-30::mNG::3xFLAG::AID levels in individual GABA MNs in ethanol-treated (control) or 4 mM auxin-treated animals for 2 days, from L3 to adult day 1 (Fig. 7B). Compared to ethanol-treated animals, auxin-treated animals showed a robust reduction in the levels of UNC-30::mNG::3xFLAG::AID fluorescence intensity, indicating efficient depletion (Fig. 7C-E). Auxin-treated animals also exhibited a significant reduction in *mScarlet::madd-4B* fluorescence intensity levels in all nerve cord GABAergic MNs (Fig. 7F-H, Fig. S6). Hence, UNC-30 is required during late larval and young adult stages to maintain *madd-4B* expression in GABAergic MNs (Fig. 7I). The continuous requirement of UNC-30 is likely to be essential to maintain GABA_A_R clustering throughout life.

### UNC-30 is required to maintain expression of GABA biosynthesis genes

**Fig. 8.**
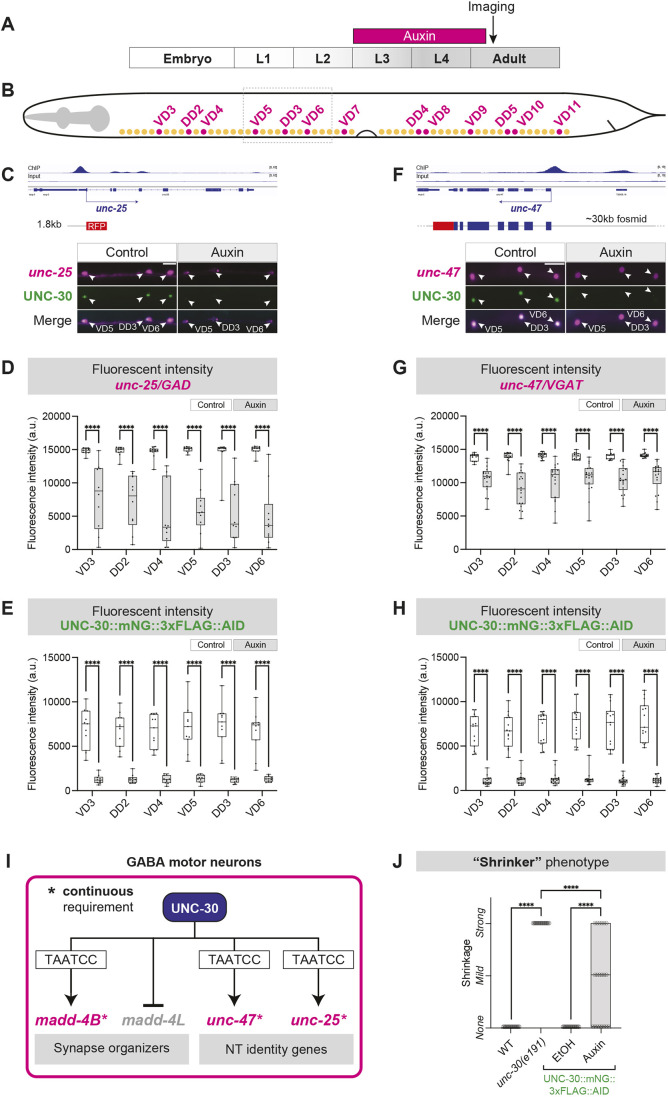
**UNC-30 is required to maintain expression of GABA biosynthesis genes.** (A) Auxin treatment timeline. Day 1 adults were imaged. (B) Schematic of *C. elegans* with dashed box depicting the imaged area shown in C and F. (C) Top: UNC-30 ChIP-seq track on the *unc-25* locus. Bottom: Expression of *unc-25(hpIs88 [unc-25p::mCherry])* and UNC-30 (*syb2344 [UNC-30::mNG::3xFLAG::AID]*) in control (EtOH treated) and auxin-treated animals. Animals express TIR1 pan-somatically (*ieSi57[Peft-3::TIR1::mRuby]*). White arrowheads indicate GABAergic MNs. and *unc-47* (F) loci. Scale bar: 10 µm. (D,E) Quantification of *unc-25(hpIs88 [unc-25p::mCherry])* or UNC-30 (*syb2344 [UNC-30::mNG::3xFLAG::AID]*) fluorescence intensity. Two-way ANOVA followed by Sidak's multiple comparison test. ****P<0.0001. Control: *n*=11; auxin treated: *n*=10. (F) Top: UNC-30 ChIP-seq tracks on the *unc-47* locus. Bottom: Expression of *unc-47* (*otIs565 [unc-47(fosmid)::SL2::H2B::mChopti]*) and UNC-30 (*syb2344 [UNC-30::mNG::3xFLAG::AID]*) in control (EtOH) and auxin-treated animals expressing TIR1 pan-somatically (*ieSi57[Peft-3::TIR1::mRuby]*). Arrowheads indicate GABAergic MNs. Scale bar: 10 µm. (G,H) Quantification of *unc-47* (*otIs565 [unc-47(fosmid)::SL2::H2B::mChopti]*) or UNC-30 (*syb2344 [UNC-30::mNG::3xFLAG::AID]*) fluorescence intensity in GABAergic MNs. Two-way ANOVA followed by Sidak's multiple comparison test. ****P<0.0001. Control: *n*=15; auxin treated: *n*=18. (I) Schematic showing that UNC-30 is required to maintain *unc-25*, *unc-47* and *madd-4B* expression in GABAergic MNs. (J) Quantification of the ‘shrinker’ phenotype upon response to touch. One-way ANOVA followed by Sidak's multiple comparison test. ****P<0.0001. Wild type (WT): *n*=20; *unc-30(e191)*: *n*=20; control: *n*=20; auxin treated: *n*=20.

Prompted by our *madd-4B* observations, we next examined whether UNC-30 is continuously required to maintain the expression of additional target genes. A previous study using the strong LOF allele *e191* showed that UNC-30 activates the expression of two GABA identity genes during development: *unc-25* and *unc-47* (Eastman et al., 1999). We obtained similar results by using the same *unc-30(e191)* allele (Fig. S7). Mutating the UNC-30 binding site (TAATCC) in transgenic *unc-25* and *unc-47* reporter animals resulted in reduced reporter expression in GABA MNs, strongly suggesting UNC-30 regulates these targets via direct binding (Eastman et al., 1999). Consistent with these previous findings, analysis of the UNC-30 ChIP-Seq dataset showed UNC-30 binding in the *cis*-regulatory regions of *unc-25* and *unc-47* endogenous loci (Fig. 8C,F).

Whether UNC-30 is required at post-embryonic stages to maintain the expression of these crucial determinants of GABAergic identity (e.g. *unc-25*, *unc-47*) and function is not known. We again employed the AID system in late larval stages, this time assessing the effect of UNC-30 depletion on expression levels of *unc-25* and *unc-47*. We observed a significant reduction in their expression levels in all nerve cord GABAergic MNs (Fig. 8A-H, Fig. S8), suggesting that UNC-30 is not only required during early development to initiate expression of GABA biosynthesis genes, but also to maintain their expression during late larval stages (Fig. 8I).

### UNC-30 is continuously required for normal touch response

Is UNC-30 also continuously required for normal animal behavior? Animals lacking *unc-30* gene activity (homozygous strong loss-of-function mutants) display a characteristic locomotory phenotype nicknamed ‘shrinker’ (McIntire et al., 1993; Chalfie and Jorgensen, 1998), i.e. *unc-30* mutants hypercontract their body wall muscles in response to touch owing to the lack of GABAergic MN inhibitory input to muscles. We indeed observed a striking and fully penetrant ‘shrinker’ phenotype in *unc-30(e191)* mutants compared with control animals (Fig. 8J). Importantly, auxin-mediated depletion of UNC-30 specifically at late larval/early adult stages also resulted in ‘shrinker’ animals (Fig. 8J). Because the auxin system does not fully eliminate UNC-30, as evidenced by quantification of UNC-30::mNG::3xFLAG::AID expression levels in individual GABAergic MNs (Fig. 7H), the ‘shrinker’ phenotype displayed variable expressivity (none, mild, strong) upon auxin treatment (Fig. 8J). In the control (ethanol) condition, we observed no shrinkers, suggesting that tagging the endogenous *unc-30* gene with the mNG::3xFLAG::AID cassette does not result in detectable hypomorphic effects on locomotory behavior (Fig. 8J). We conclude that UNC-30 is continuously required for normal touch response.

### The dual role of UNC-30 in GABA MNs extends to other target genes

A handful of UNC-30 target genes are known to date, including *unc-25*, *unc-47*, *pde-4/PDE4B*, *acy-1/ADCy9*, *oig-1*, *flp-11*, *flp-13* and *ser-2* (Table 1) (Cinar et al., 2005; Eastman et al., 1999; Howell et al., 2015; Shan et al., 2005; Yu et al., 2017). A unifying theme emerging from these studies is that UNC-30 acts as a transcriptional activator. Our findings on *madd-4L* (Fig. 6), however, suggest a repressive role for UNC-30 in GABA MNs. We therefore sought to identify new UNC-30 target genes to determine whether the duality in UNC-30 function (activator and repressor) is broadly employed.

**Table 1.**
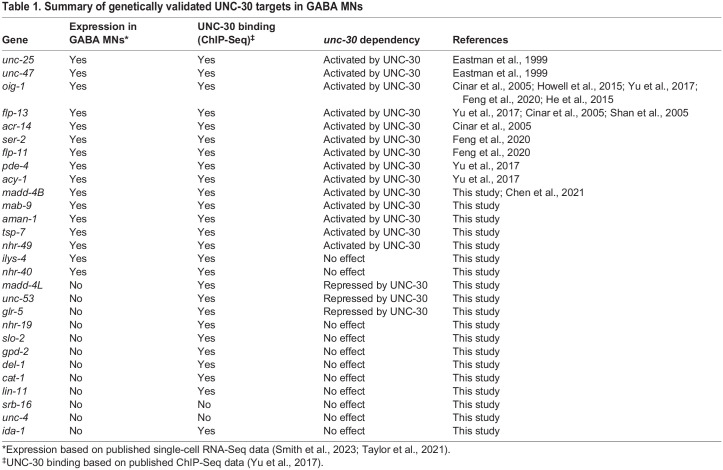
Summary of genetically validated UNC-30 targets in GABA MNs

**Fig. 9.**
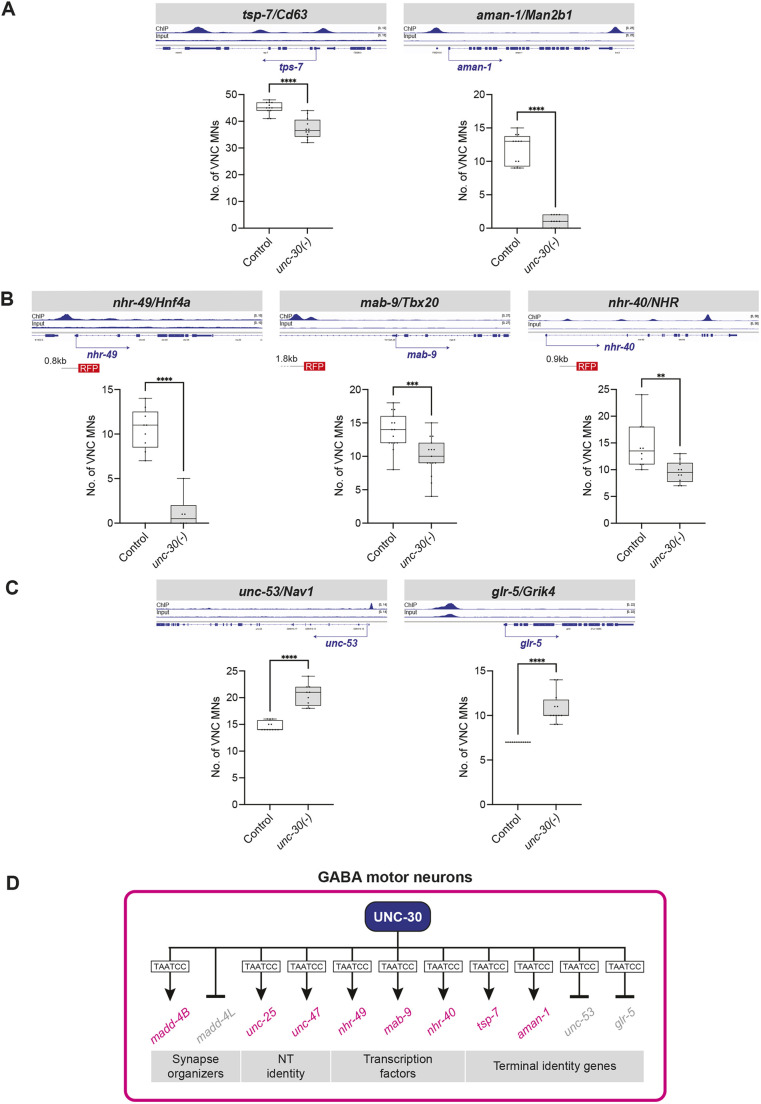
**UNC-30 activates and represses different genes in GABAergic MNs.** (A-C) Quantification of the number of GABAergic MNs expressing *wdEx351 [tsp-7::GFP], sEx11477 [aman-1::GFP])*, *kasEx220 [nhr-49 (−803 to +58 bp from ATG)::RFP]*, *kasEx232 [mab-9 (−5569 to −3768 bp from ATG)::RFP]*, *kasEx214 [nhr-40(+938 to +1846 bp from ATG)::RFP]*, *hdIs1 [unc-53p::GFP]* or *icIs270 [glr-5::GFP]* in control and *unc-30(191)* animals. Stage: L4. Box and whisker plots show median, and lower and upper quartiles; whiskers represent minimum and maximum. Black circles depict individual values. Unpaired *t*-test with Welch's correction. **P<0.002, ***P<0.0002, ****P<0.0001. Wild type (control): *n*=10; *unc-30(e191)*: *n*=10. (D) Summary of UNC-30 targets in GABAergic motor neurons. The canonical UNC-30 binding site (TAATCC) is shown, although it was not experimentally validated for all genes.

First, we identified putative *unc-30* targets by searching for UNC-30 binding peaks in genes that are normally expressed in GABA MNs (Smith et al., 2024; Taylor et al., 2021) (Table 1). In total, we tested six genes (*tsp-7/Cd63*, *aman-1/Man2b1*, *nhr-49/Hnf4a*, *mab-9/Tbx20*, *nhr-40/NHR*, *ilys-4*) by either generating new transgenic reporter animals (*nhr-49*, *mab-9*, *nhr-40*), or using available reporters (*tsp-7*, *aman-1*, *ilys-4*). Reporter expression for five of these genes (*tsp-7*, *aman-1*, *nhr-49*, *mab-9*, *nhr-40*) was significantly reduced in GABA MNs of *unc-30 (e191)* mutant animals (Fig. 9A,B, Table 1). Because ChIP-Seq shows UNC-30 binding to all four of these genes (Fig. 9A,B), it is likely that UNC-30 acts as a direct activator of *tsp-7*, *aman-1*, *nhr-49*, *nhr-40* and *mab-9* transcription (Fig. 9D).

Next, we aimed to identify genes that, like *madd-4L*, are repressed by UNC-30. We searched for UNC-30 binding peaks in genes that are not expressed in GABA MNs, but instead are normally expressed in cholinergic nerve cord MNs (Table 1). In total, we tested 11 genes, for which transgenic reporter animals were available. Two (*unc-53*/*NAV1* and *glr-5*/*GRIK4*) of the 11 reporters showed ectopic expression in GABA MNs of *unc-30(e191)* mutant animals (Fig. 9C, Table 1). Interestingly, late larval depletion of UNC-30 with the AID system did not affect *glr-5* expression, suggesting UNC-30 is not required at later stages to repress this gene in GABA MNs (Fig. S9).

Altogether, our work identified nine new UNC-30 target genes; six are activated (*madd-4B, tsp-7*, *aman-1*, *nhr-49*, *mab-9*, *nhr-40*) and three are repressed (*madd-4L*, *unc-53*, *glr-5*) by UNC-30 (Fig. 9D). This analysis significantly expands the known repertoire of UNC-30 target genes in the *C. elegans* nervous system (Table 1), consolidating its previously known activator role and uncovering a putative repressive function.

## DISCUSSION

### Transcriptional coordination of NT biosynthesis in the presynaptic cell and postsynaptic NT receptor clustering

Here, we describe a molecular mechanism that coordinates two spatially separated processes that are essential for the function of chemical synapses: NT biosynthesis in the presynaptic cell and NT receptor clustering at the postsynaptic cell. Using *C. elegans* neuromuscular synapses as a model, we show that the terminal selector-type transcription factor UNC-30 is required continuously to maintain expression of GABA identity genes (e.g. *unc-25*, *unc-47*) in presynaptic GABAergic MNs, thereby ensuring GABA synthesis and release. In postsynaptic target muscle cells, UNC-30 acts non-cell-autonomously to control clustering of GABA_A_Rs – the most prominent inhibitory NT receptors in animal nervous systems (Ghit et al., 2021; Goetz et al., 2007). Mechanistically, we propose that UNC-30 directly regulates the production of MADD-4B, a secreted synapse organizer. Hence, UNC-30 coordinates GABA_A_R clustering on postsynaptic muscle cells with the acquisition and maintenance of GABAergic identity of presynaptic cells (Fig. 1C, model 3), safeguarding GABA neurotransmission.

UNC-30 is required in late larval and adult stages to maintain expression of GABA biosynthesis genes (e.g. *unc-25*, *unc-47*), consolidating its role as a terminal selector of GABA MN identity (Eastman et al., 1999; Jin et al., 1994). Further, UNC-30 acts directly to activate and maintain transcription of *madd-4B*, a secreted synapse organizer necessary for GABA_A_R clustering on target muscle cells (Pinan-Lucarre et al., 2014). Because UNC-30 is continuously required in GABA MNs, this simple co-regulatory strategy of *unc-25*, *unc-47* and *madd-4B* by a terminal selector may ensure that key features of a functional synapse will continue to appear together throughout life (Fig. 1C). Hence, the presynaptic neuron will continue to synthesize and release GABA (ensured by continuous *unc-25* and *unc-47* expression) and the postsynaptic neuron will constantly have the means to receive GABA via cognate receptor clustering (ensured by continuous *madd-4B* expression). Because UNC-30 orthologs are expressed in planarian (Currie and Pearson, 2013; Marz et al., 2013), fly (Vorbruggen et al., 1997), zebrafish (Shi et al., 2005) and mouse (Bifsha et al., 2017; Luk et al., 2013) nervous systems, the co-regulatory principle described here may be broadly applicable across species.

### Terminal selectors control synaptic connectivity

The only other known example of a terminal selector that operates in an analogous manner is UNC-3, the sole *C. elegans* ortholog of the COE (Collier/Olf/EBF) family of proteins (Dubois and Vincent, 2001). In nerve cord cholinergic MNs, UNC-3 acts as a terminal selector, directly regulating scores of effector genes (e.g. ACh biosynthesis proteins, ion channels) (Kratsios et al., 2012; Li et al., 2020). Like *unc-30*, *unc-3* is not expressed in *C. elegans* muscles, yet it is required for AChR clustering on muscle cells (Kratsios et al., 2015). In cholinergic MNs, UNC-3 not only directly activates *madd-4B* (function of which in these cells is discussed in the next section), but also *madd-4L*, which is required for AChR clustering (Kratsios et al., 2015). By contrast, we find that UNC-30 activates *madd-4B* but represses *madd-4L* in GABA MNs, thereby ensuring expression of the appropriate *madd-4* isoform (*madd-4B*). Altogether, NT receptor clustering in *C. elegans* neuromuscular synapses is achieved by two different terminal selectors regulating, in distinct ways, the two isoforms of the same synapse-organizing molecule: UNC-3 activates both *madd-4B* and *madd-4L* in cholinergic MNs, whereas UNC-30 activates *madd-4B* but represses *madd-4L* in GABA MNs.

Besides NT receptor clustering, additional synaptic connectivity defects have been reported in MNs of *unc-3* and *unc-30* mutant animals (Barbagallo et al., 2017; He et al., 2015; Howell et al., 2015; Kratsios et al., 2015; Philbrook et al., 2018). Specifically, cholinergic MN input onto GABA MNs is disrupted in *unc-3* mutants (Barbagallo et al., 2017). In this case, UNC-3 controls *nrx-1/*neurexin, a synapse organizer necessary for AChR localization onto dendrites of GABA MNs (Philbrook et al., 2018). UNC-30 has been implicated in *C. elegans* synaptic remodeling, as it is necessary to prevent premature synapse rewiring of DD neurons and aberrant synapse rewiring of VD neurons (He et al., 2015; Howell et al., 2015). This is achieved by UNC-30 directly regulating OIG-1, a single immunoglobulin domain protein that functions as a synaptic organizer (He et al., 2015; Howell et al., 2015). Consistent with a recent review (Hobert and Kratsios, 2019), this work and the aforementioned studies provide strong evidence for expanding the definition of terminal selector genes; they not only regulate effector genes required for NT biosynthesis and neuronal signaling (e.g. ion channels), but also control synaptic connectivity via the regulation of distinct synapse organizers.

It is tempting to speculate that mammalian terminal selectors may operate in an analogous manner. For example, the terminal selector of mouse spinal MNs, Isl1 (Cho et al., 2014), may control transcription of agrin, an MN-derived synapse organizer necessary for AChR clustering in mouse skeletal muscles (Sanes and Lichtman, 1999).

### Neuron type-specific regulation of synapse organizers

Synapse organizers are cell adhesion or secreted molecules that control synapse formation and/or maintenance (Johnson-Venkatesh and Umemori, 2010; Mizumoto et al., 2023). Their adhesive and signaling properties mediate uni- or bidirectional signaling, enabling pre- and/or postsynaptic differentiation (Sanes and Zipursky, 2020). Understanding the spatiotemporal regulation of synapse organizers is important because synapses must be built at the right place and time. However, we know very little about the transcriptional mechanisms that control synapse organizer expression, in part because these molecules usually have multiple isoforms (e.g. neurexins, neuroligins, agrin, MADD-4/punctin) (Fukai and Yoshida, 2021; Gomez et al., 2021; Yamagata et al., 2015). Multiple isoforms can be produced via either alternative RNA splicing or promoter usage. To date, substantial research has focused on alternative splicing of synapse organizers (e.g. neurexin isoforms) (Traunmuller et al., 2023), leaving their transcriptional mechanisms poorly understood.

MADD-4L is only produced by cholinergic MNs (Kratsios et al., 2015; Pinan-Lucarre et al., 2014). Upon secretion, it promotes clustering of L-AChRs by an extracellular scaffold composed of LEV-10 (LEVamisole resistant-10), LEV-9 and OIG-4 (One ImmunoGlobulin domain-4) (Gally et al., 2004; Gendrel et al., 2009; Pinan-Lucarre et al., 2014; Rapti et al., 2011). By contrast, MADD-4B is produced by both cholinergic and GABAergic MNs. At GABAergic neuromuscular synapses, MADD-4B promotes GABA_A_R clustering on muscle cells through binding to NLG-1/neuroligin and activation of UNC-40/DCC signaling (Zhou et al., 2020; Tu et al., 2015; Maro et al., 2015). At cholinergic neuromuscular synapses, MADD-4B inhibits the attraction of GABA_A_ receptors by MADD-4L (Pinan-Lucarre et al., 2014). Hence, spatial (neuron type-specific) regulation of MADD-4 isoform expression is crucial for the formation and function of excitatory (ACh) and inhibitory (GABA) synapses in *C. elegans.* Our previous work identified UNC-3 as a critical activator of both *madd-4* isoforms in cholinergic MNs (Kratsios et al., 2015). Here, we show in GABA MNs that UNC-30 controls the two *madd-4* isoforms in opposite ways; it provides direct and positive input to the *madd-4B* promoter and negative input to the *madd-4L* promoter, thereby ensuring proper GABA_A_R clustering on target muscle cells.

### Advancing our understanding of PITX gene function in the nervous system

In humans, PITX gene mutations cause various congenital defects and cancer (Tran and Kioussi, 2021). Pitx genes belong to the PAIRED (PRD) class of highly conserved homeobox genes. In mice, Pitx genes play crucial roles in the development of the nervous system, craniofacial structures, and limbs (reviewed by Tran and Kioussi, 2021). *Pitx2* and *Pitx3* are expressed in discrete cell populations of the mouse midbrain and spinal cord (Bifsha et al., 2017; Luk et al., 2013; Zagoraiou et al., 2009). *Pitx3* is essential for the survival of dopaminergic neurons of the substantia nigra, a key cellular substrate of Parkinson's disease (Luk et al., 2013). Importantly, human variants of *PITX2* or *PITX3* affect eye development (Tran and Kioussi, 2021; Semina et al., 1998). *PITX2* variants cause Axenfeld–Rieger syndrome, a disorder that affects primarily the eyes, whereas PITX3 variants are associated with congenital cataracts (Muzyka et al., 2023; Tumer and Bach-Holm, 2009).

Mechanistically, functional assays showed that human *PITX2* and *PITX3* gene variants result in reduced transcriptional activity (Wang et al., 2013; Zhao et al., 2015; Verdin et al., 2014). However, the transcriptional targets of PITX proteins remain poorly defined and whether they act as transcriptional activators and/or repressors is not well defined. Our study contributes to these knowledge gaps in three ways. First, we identify nine new UNC-30 target genes (activated: *madd-4B*, *mab-9*, *nhr-49*, *nhr-40*, *tsp-7*, *aman-1*; repressed: *madd-4L*, *unc-53*, *glr-5*), significantly expanding the list of PITX targets in the nervous system (Table 1). Second, consistent with its previously described direct mode of activation of genes involved in GABA biosynthesis and neuronal rewiring (Eastman et al., 1999; Yu et al., 2017; Howell et al., 2015; He et al., 2015), our mutational analysis indicates that UNC-30 acts directly to activate *madd-4B*. Last, we propose that, in GABA MNs, UNC-30 acts both as an activator and repressor of distinct sets of genes. A similar dual role for UNC-30 has recently been described in *C. elegans* glia, where it promotes GLR glia morphology and represses alternative mesodermal fates (Stefanakis et al., 2024).

### Limitations of this work

Future studies are needed to dissect the molecular mechanism underlying the dual role of UNC-30 in GABA MNs. It is likely that cooperation with distinct transcription factors shifts its transcriptional activity from an activator to a repressor. Candidates include LIN-39/HOX, a known transcriptional activator in GABA MNs (Feng et al., 2020), and UNC-55/NR2F, a known transcriptional repressor in these cells (Shan et al., 2005; Yu et al., 2017). Another limitation relates to maintenance of GABA_A_R clustering. Although we showed that UNC-30 is required to maintain *madd-4B* transcription in late larval/early adult stages, it remains unknown whether inducible UNC-30 depletion at these stages affects maintenance of GABA_A_R clustering. Alternatively, it is possible that MADD-4B secretion is only required to initiate GABA_A_R clustering, but subsequent maintenance of clustering may rely on other factors intrinsic to the postsynaptic cell.

Although GABA_A_Rs undergo similar ectopic clustering at cholinergic neuromuscular junctions in both *madd-4(tr185)* and *unc-30(e191)* animals, there is a difference: GABA_A_Rs partially cluster at GABAergic neuromuscular junctions in the *madd-4(tr185)* mutant, whereas they fail to properly cluster at these synapses in the *unc-30(e191)* mutant (Figs 2 and 4). This suggests that, in addition to *madd-4B*, other yet-to-be identified UNC-30 target genes may also act as GABA synapse organizers. In the *madd-4(tr185)* mutant, these additional genes may operate in parallel with MADD-4L, which has been shown to trap GABA_A_Rs at cholinergic neuromuscular junctions (Pinan-Lucarre et al., 2014). Last, our work is focused on neuromuscular synapses. Notably, punctin (ADAMTSL3) and other secreted synapse organizers (e.g. cerebellins, pentraxins, Sema3F, BDNF) are expressed in the mammalian brain (Dow et al., 2011; Uemura et al., 2010; Sigoillot et al., 2015; Cramer et al., 2023). Hence, similar co-regulatory strategies to the one described here may operate in neuron–neuron or neuron–glia synapses in the central nervous system.

## MATERIALS AND METHODS

### *C. elegans* strains

Worms were grown at 15°C, 20°C or 25°C on nematode growth media (NGM) plates seeded with bacteria (*Escherichia coli* OP50) as food source. All *C. elegans* strains used in this study are listed in Table S1.

### Generation of transgenic reporter animals

Reporter gene fusions for *cis*-regulatory analysis were made using either PCR fusion or Gibson Assembly Cloning Kit (NEB, 5510S) (Hobert, 2002). Targeted DNA fragments were fused (ligated) to *tagrfp* or *gfp* coding sequence, followed by the *unc-54* 3′ UTR*.* Mutations of UNC-30 binding sites were introduced by PCR mutagenesis. The product DNA fragments were either injected into young adult *pha-1(e2123)* hermaphrodites at 50 ng/µl using *pha-1* (pBX plasmid) as co-injection marker (50 ng/µl) and further selected for survival, or injected into young adult N2 hermaphrodites at 50 ng/µl (plus 50 ng/µl pBX plasmid) using *myo-2::gfp* as co-injection marker (3 ng/µl) and further selected for GFP signal. Primer sequences used for reporter construct generation are provided in Table S2.

### Generation of single-copy insertion alleles

Single-copy insertion alleles *krSi92 [Punc-47::T7::madd-4S::GFP]* and *krSi342 [Punc-30::gfp1-10]* were generated by the miniMos method (Frokjaer-Jensen et al., 2014). Worms were injected with 15 ng/μl plasmid of interest containing the promoter and the open reading frame fused to fluorescent proteins, 50 ng/μl pCFJ601 (Mos1 transposase), 10 ng/μl pMA122 (negative selective marker *Phsp16.2::peel-1*) and 2.5 ng/μl pCFJ90 (*Pmyo-2::mCherry*). Neomycin (G418) was added to plates 24 h after injection at 1.5 μg/μl final concentration. Candidate plates were heat-shocked for 2 h at 34°C. Worms with an insertion were isolated and subsequently maintained as homozygous carriers of *krSi92* or *krSi342*.

### Targeted genome engineering

CRISPR/Cas9 genome editing was performed by SunyBiotech or SEGICel following standard procedures (Dickinson and Goldstein, 2016). The *unc-30* endogenous reporter allele *syb2344 [unc-30::mNG::3xFlag::AID]* was generated by SunyBiotech via CRISPR/Cas9 by inserting the *mNG::3xFLAG::AID* cassette immediately before the *unc-30* termination codon. The endogenous *madd-4L* reporter allele *syb624 [2xNLS::mScarlet::SL2::madd-4L]* was generated by inserting the *2xNLS::mScarlet::SL2* cassette immediately after the ATG of *madd-4L*. The endogenous *madd-4B* reporter allele *syb623 [2xNLS::mScarlet::SL2::madd-4B]* was generated by inserting the *2xNLS::mScarlet::SL2* cassette immediately after the ATG of *madd-4B*. The *mScarlet* sequence is preceded by two copies of a nuclear localization signal (2xNLS) and followed by the SL2 trans-splicing element (Fig. 5A). Hence, the *2xNLS::mScarlet* sequence and endogenous *madd-4B* are transcribed as one mRNA, but each is translated independently as a result of the SL2 element. The endogenous *madd-4B* reporter allele *syb3561 [2xNLS::mScarlet::SL2::madd-4B*^Δ506 bp^*]* was generated by creating a 506 bp-long deletion (−1433 bp to −927 bp from the *madd-4B* ATG) in the background strain carrying the endogenous *madd-4B* reporter allele *syb623 [2xNLS::mScarlet::SL2::madd-4B].* The endogenous *madd-4L* reporter alleles *kas31 and kas32* were generated by mutating the canonical UNC-30 binding site I (TAATCC) to GCGCGC in the background strain carrying the endogenous *madd-4L* reporter allele *syb624 [2xNLS::mScarlet::SL2::madd-4L]*.

The *cla-1 bab462 [cla-1::spgfp11×7]* knock-in allele was generated by inserting the *spgfp11×7* cassette immediately before the STOP of *cla-1*.

### Temporally controlled protein degradation

AID-tagged proteins are conditionally degraded when exposed to auxin in the presence of TIR1 (Ashley et al., 2021; Zhang et al., 2015). Animals carrying auxin-inducible alleles of *unc-30 (syb2344[unc-30::mNG::3xFLAG::AID]) IV* were crossed with *ieSi57* animals that express TIR1 pan-somatically. Auxin (indole-3-acetic acid; Alfa Aesar, A10556) was dissolved in ethanol (EtOH) to prepare 400 mM stock solutions which were stored at 4°C for up to 1 month. NGM agar plates were poured with auxin or EtOH added to a final concentration of 4 mM and allowed to dry overnight at room temperature. Plates were seeded with OP50 bacteria. To induce protein degradation, worms of the experimental strains were transferred onto auxin-coated plates and kept at 20°C. As a control, worms were transferred onto EtOH-coated plates instead. Auxin solutions, auxin-coated plates and experimental plates were shielded from light.

### Microscopy

For Figs 2-4 and  Fig. S1, young adult *C. elegans* were mounted on 2% agarose (w/v in water) dry pads immersed in 2% polystyrene beads (0.1 mm diameter, Polyscience, 00876-15) diluted in M9 buffer. Images were taken using a Nikon-IX86 microscope (Olympus) equipped with an Andor spinning disk system (Oxford Instruments), a 60×/NA 1.42 oil immersion objective and an Evolve EMCCD camera. For each animal (Figs 2-4, Fig. S1), an image of the DNC or VNC at the first quarter of the worm was acquired as a stack of optical sections (0.2 µm apart). Pearson's coefficient was calculated as described (Tu et al., 2015). Pearson's r values are indicated. For the remaining figures, worms were anesthetized using 100 mM sodium azide (NaN_3_) and mounted on a 4% agarose pad on glass slides. Images were taken using an automated fluorescence microscope (Zeiss, Axio Imager.Z2). Several *z*-stack images (each ∼1 µm thick) were acquired with a Zeiss Axiocam 503 mono using the ZEN software (Version 2.3.69.1000, Blue edition). Representative images are shown following maximum projection of 1-8 µm *z*-stacks using the maximum intensity projection type. Image reconstruction was performed using ImageJ/Fiji software (Schindelin et al., 2012).

### MN identification

MNs were identified based on a combination of the following factors: (1) colocalization with fluorescent markers with known expression pattern, (2) invariant cell body position along the VNC, or relative to other MN subtypes, (3) MN birth order, and (4) number of MNs that belong to each subtype.

### Immunofluorescence staining

For Fig. S1, immunofluorescence staining was performed as described (Tu et al., 2015). Images were acquired using a Leica 5000B microscope equipped with a spinning disk CSU10 (Yokogawa) and a Coolsnap HQ2 camera.

### Fluorescence intensity (FI) quantification

To quantify FI of individual MNs in the VNC, images of worms from different genetic backgrounds were taken with identical parameters through full-thickness *z*-stacks that covered the entire cell body. Image stacks were then processed and quantified for FI using Fiji. The focal plane in *z*-stacks that had the brightest FI was selected for quantification. Background signal was minimized by using Fiji's background subtraction feature (rolling ball at 50 pixels). The cell outline was manually selected, and Fiji was used to quantify the FI and area to get the mean value for FI.

### Statistical analysis and reproducibility

For quantification, box and whisker plots were adopted to represent the quartiles in graphs. The box includes data points from the first to the third quartile value with the horizontal line in box representing the median value. Upper and lower limits indicate the maximum and minimum, respectively. Unpaired *t*-test with Welch's correction was performed and *P*-values were annotated. Visualization of data and *P*-value calculation were performed using GraphPad Prism Version 9.2.0 (283). Each experiment was repeated twice.

For experiments presented in Figs 2-4 and Fig. S1, box and whisker plots show median, lower and upper quartiles, and whiskers represent s.d. For Pearson's correlation coefficient, fluorescence intensity and boutons density quantifications, Mann–Whitney tests (comparison of two genotypes) and Kruskal–Wallis followed by Dunn's post-tests (comparison of more than two genotypes) were performed if data did not follow a normal distribution (Shapiro normality test) or did not show equality of variances (Bartlett test). If data were normal and showed equality of variances, unpaired, two-tailed Student's *t*-tests were performed.

In all figures, box and whisker plots show median, lower and upper quartiles, whiskers represent minimum and maximum. Black circles depict values.

## Supplementary Material



10.1242/develop.202733_sup1 Supplementary information

Table S1. List of *C. elegans* strains used in this study.

Table S2. List of primers used in this study.
