# Supplementary material for: UNC-30/PITX coordinates neurotransmitter identity with postsynaptic GABA receptor clustering
Source: Development. 2024 Aug 27;151(16):dev202733. doi: 10.1242/dev.202733 (PMC11385328; doi:10.1242/dev.202733)
Supplement: Supplementary information [file develop-151-202733-s1.pdf]

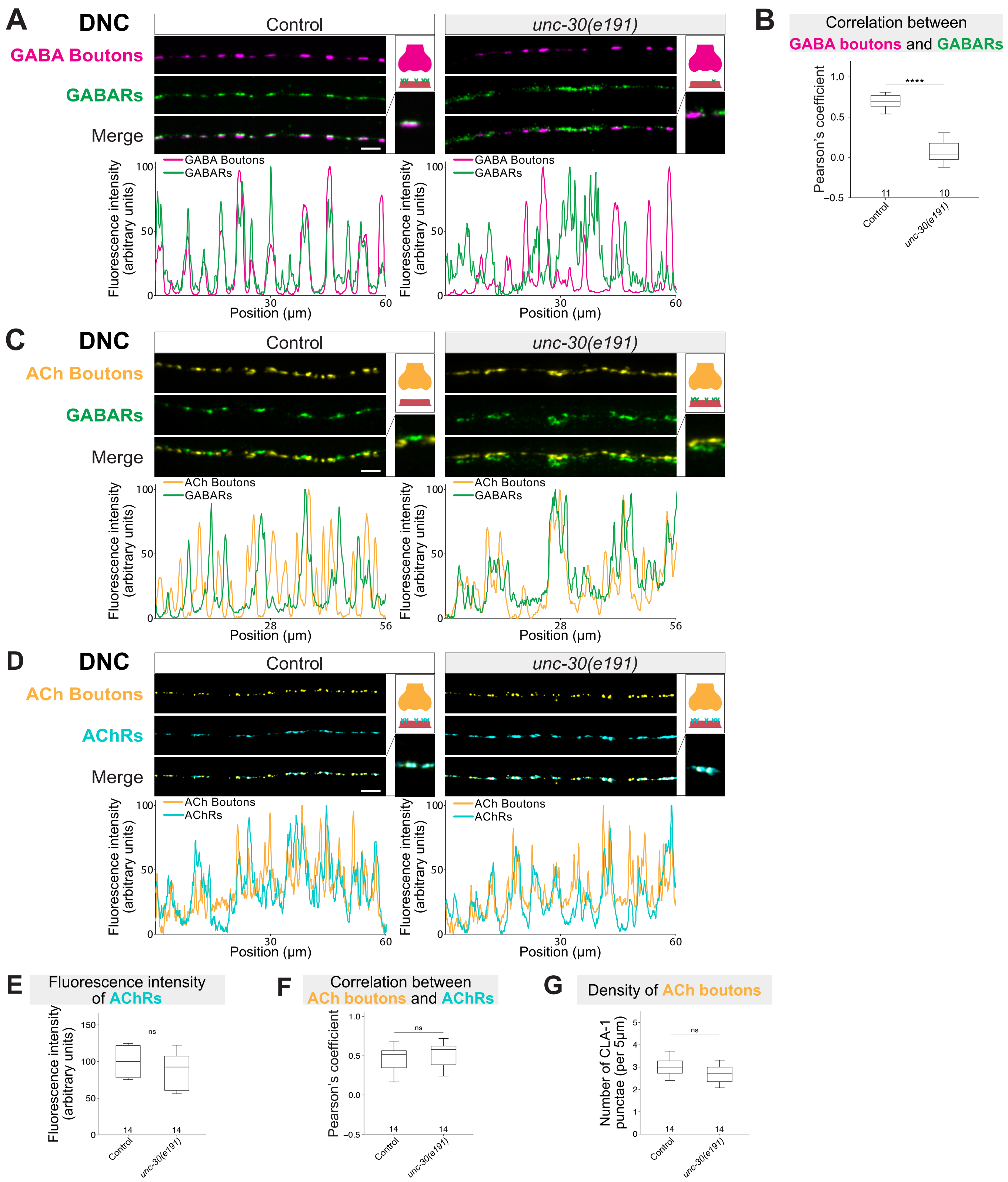

Fig. S1. Cholinergic neuromusc ular synapses in *unc-30* mutants. Related to Fig 3. (A) Fluorescence micrographs of GABA boutons (*otEx5663[unc-30prom::rab-3::gfp]*, magenta) and of GABAARs (*kr296[unc-49::rfp]*, green) in control and *unc-30(e191)* animals. Fluorescence profiles provided below. **(B)** Pearson's correlation coefficient between UNC-49-RFP and RAB-3-GFP from data shown in panel a. Mann-Whitney test. ****p<0.0001. **(C)** Immunofluorescence staining of cholinergic presynaptic boutons (anti-UNC-17/Vesicular Acetylcholine Transporter antibodies, yellow) and GABAARs (anti-UNC-49 antibodies, green) in the control (N2 strain) and unc-30(e191) animals. Fluorescence profiles provided below. Schematics: cholinergic presynaptic boutons (yellow), body wall muscle cells (pink), and GABARs (green). **(D)** Fluorescence micrographs of cholinergic presynaptic boutons (*krSi145[unc-17prom::cla-1::BFP]*, yellow) and of AChRs (knock-in allele: *kr208[unc-29::tag-rfpT]*, cyan) in control and *unc-30(e191)* animals. Fluorescence profiles provided below. Schematics: body wall muscle cells (pink), cholinergic presynaptic boutons (yellow) and AChRs (cyan). **(E)** Fluorescence intensity of UNC-29-tagRFP from data shown in panel d. Student's t-test. ns: not significant. **(F)** Pearson's correlation coefficient between UNC-29-tagRFP and CLA-1-BFP from data shown in panel d. Mann-Whitney test. ns: not significant. **(G)** Density of CLA-1-BFP puncta from data shown in panel d. Student's t-test (d, h). ns: not significant. Box and whisker plots show median, lower, and upper quartiles; whiskers represent SD. The number of worms analyzed for each genotype is indicated on the box plots. Scale bar: 5 µm.

Development • Supplementary information

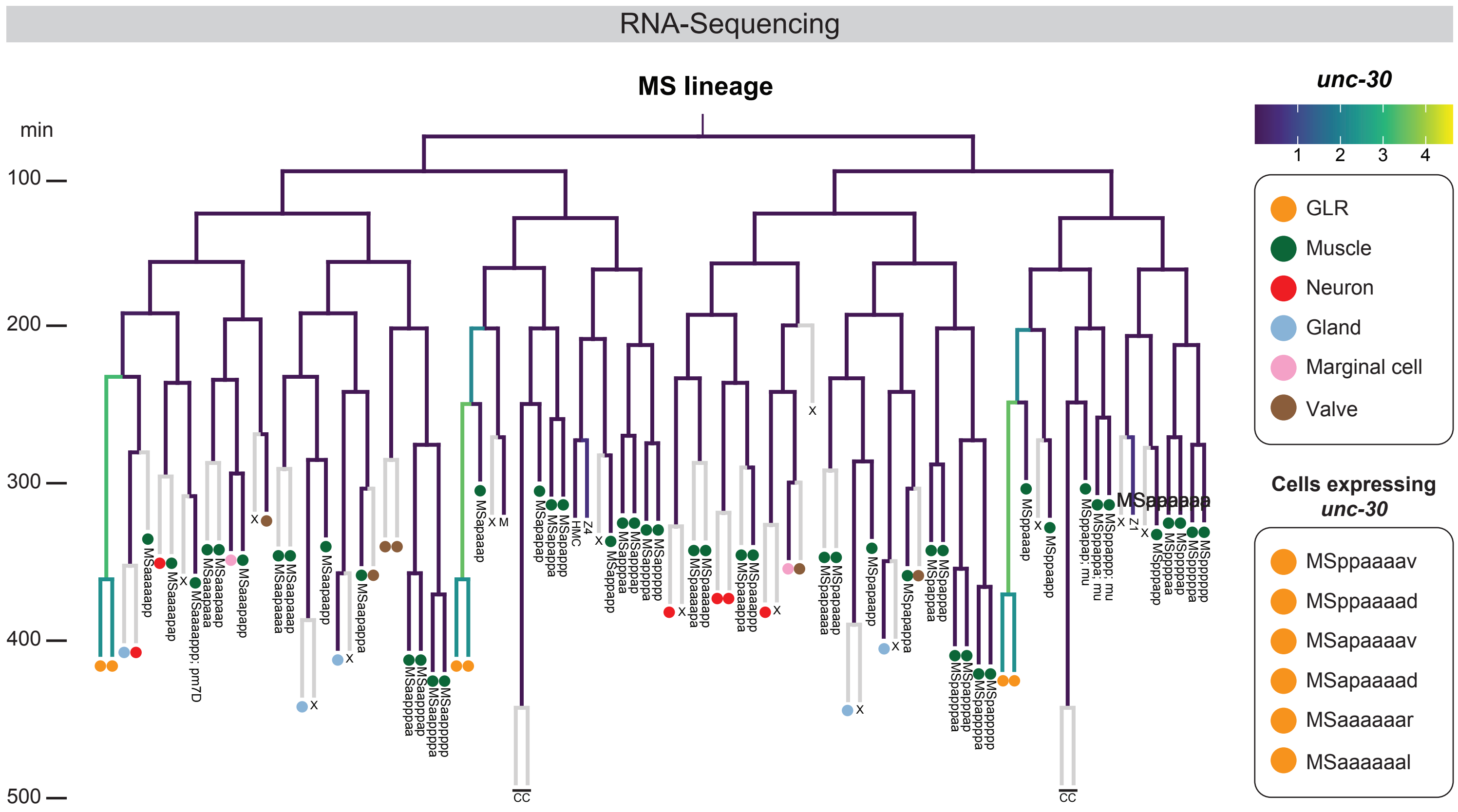

Fig. S2. *unc-30* is not expressed in *C. elegans* body wall muscle cells. Related to Figure 2. Lineage of the blast cell MS. Gradient depicts *unc-30* mRNA expression, while grey color depicts cells that were not characterized for *unc-30*. Cells expressing *unc-30* during embryogenesis are GLR glia. Single-cell RNA-Sequencing data were analyzed from Packer, J. S. et al. A lineage-resolved molecular atlas of *C. elegans* embryogenesis at single-cell resolution. *Science* 365, doi:10.1126/science.aax1971 (2019).

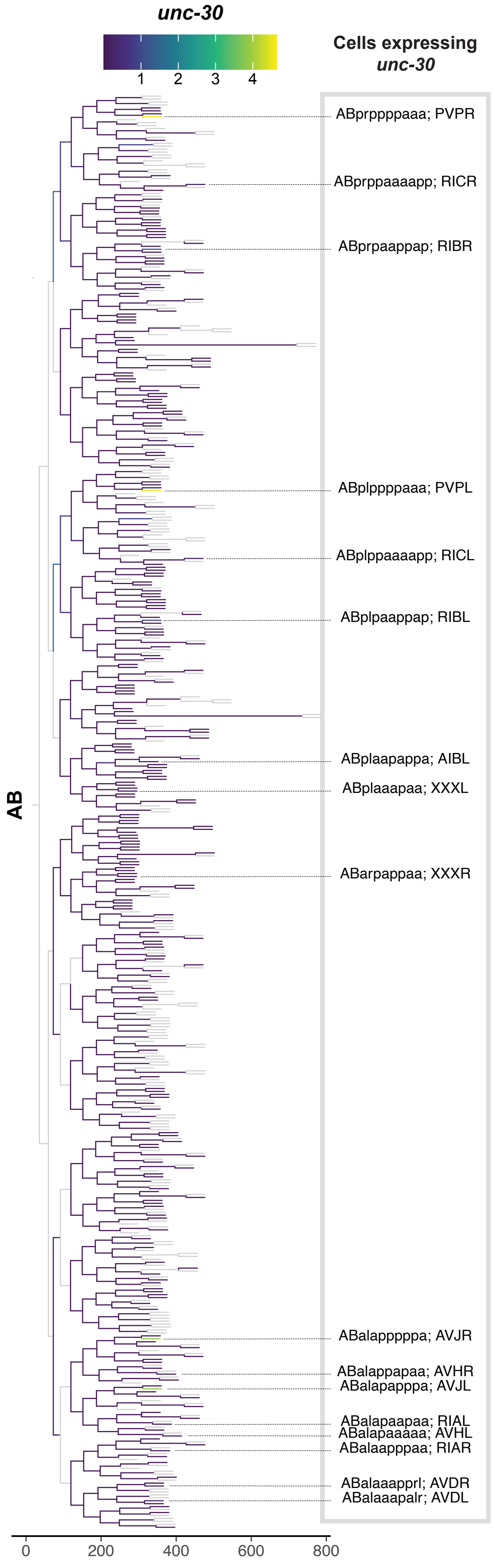

Fig. S3. Summary of *unc-30* expression in the *C. elegans* nervous system. **Related to Figure 2**. Schematic of the AB cell lineage. Gradient depicts *unc-30* mRNA expression, while grey color depicts cells that were not characterized for *unc-30*. Single-cell RNA-Sequencing data were analyzed from Packer, J. S. et al. A lineage-resolved molecular atlas of *C. elegans* embryogenesis at single-cell resolution. *Science* 365, doi:10.1126/-science.aax1971 (2019).
At the fourth larval stage (L4), *unc-30* transcripts are also detected in AVJ, PVP, DD, VD, and ASG neurons (https://cengen.shinyapps.io/CengenApp/).

Development • Supplementary information

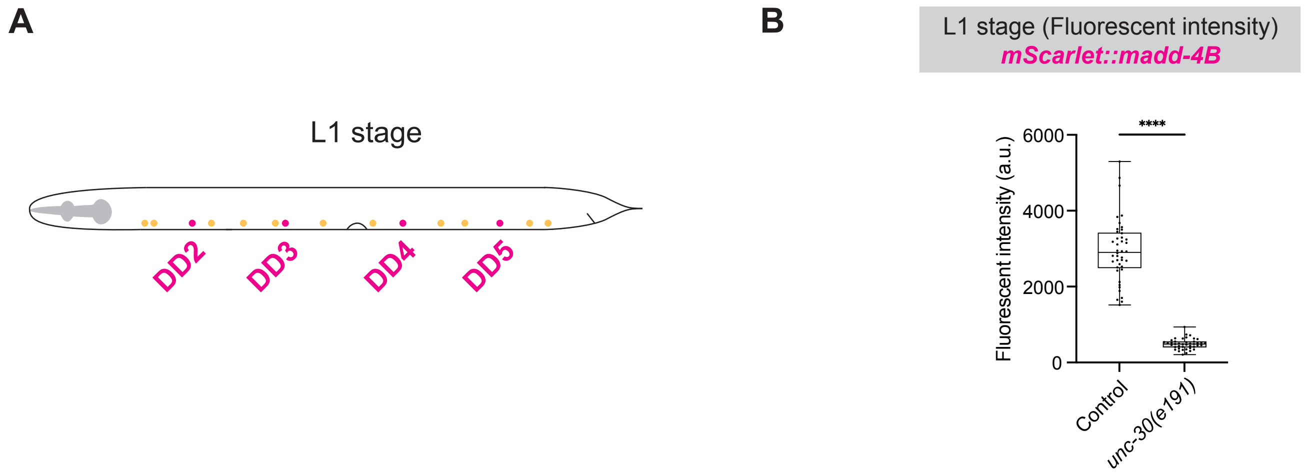

Fig. S4. UNC-30 directly activates *madd-4B* in GABAergic MNs at larval stage 1 (L1). Related to Fig. 5.
(**A**) Schematic of GABAergic (magenta) and cholinergic (yellow) nerve cord MNs at L1. MNs depicted are embryonically born.
(**B**) Quantification of *madd-4B(syb623 [2xNLS::mScarlet::SL2::madd-4B])* fluorescent intensity in GABAergic MNs at L1 in control and *unc-30(191)* animals. Animals also carry a homozygous cholinergic MN reporter (*otIs354[cho-1(fosmid)::SL2::YFP::H2B]*). Box and whisker plots show median, lower, and upper quartiles – whiskers represent minimum and maximum. Black dots depict values. Unpaired t-test with Welch's correction. ****p<0.0001. Control: n=45 MNs, *unc-30(e191)*: n=45 MNs.

Development • Supplementary information

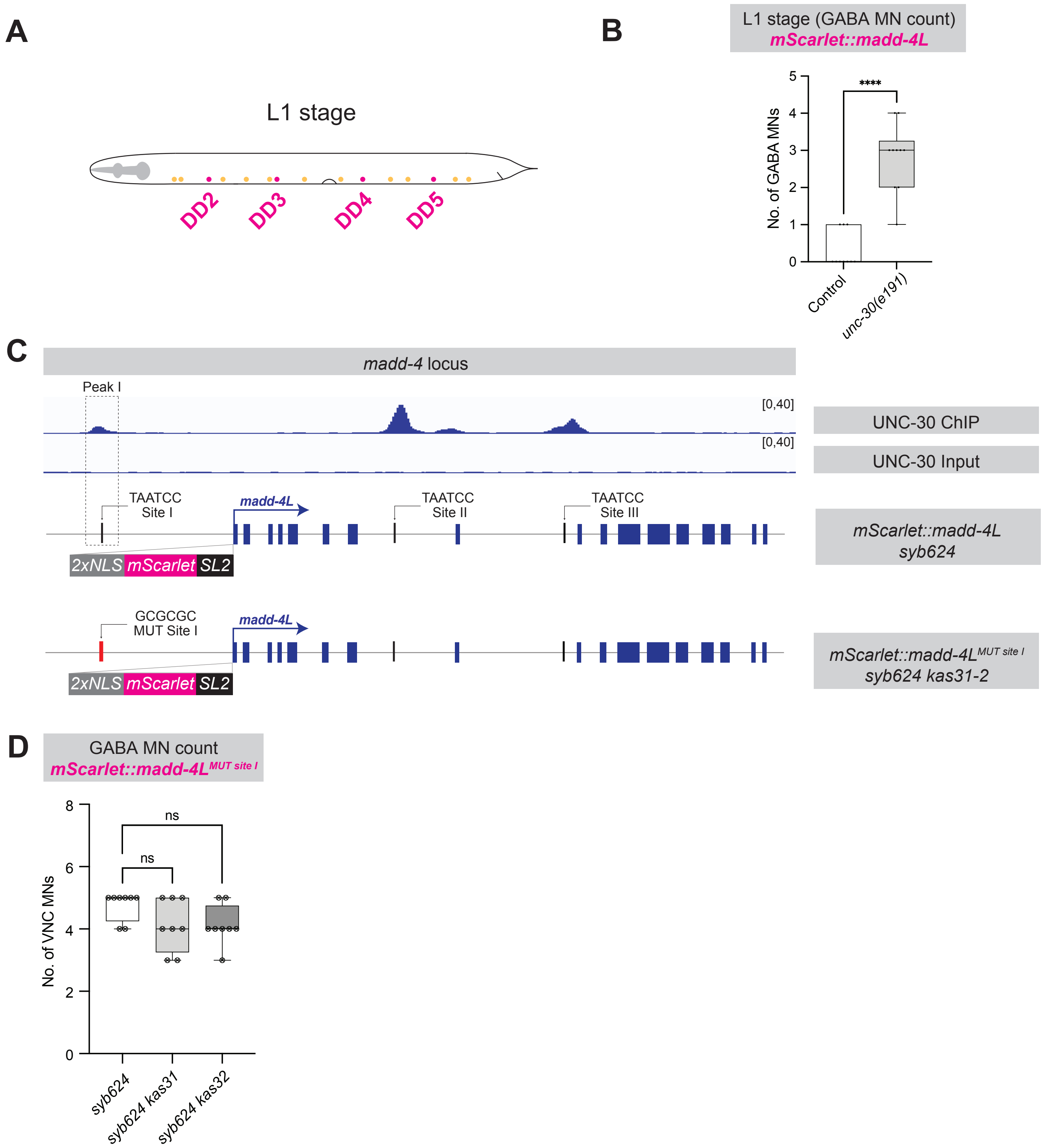

Fig. S5. UNC-30 represses *madd-4L* in GABAergic DD motor neurons at L1. Related to Fig. 6.
(**A**) Schematic of GABAergic (magenta) and cholinergic (yellow) MNs at L1. MNs depicted are embryonically born.
(**B**) Quantification of GABAergic MNs expressing *madd-4L(syb624 [2xN-LS::mScarlet::SL2::madd-4L])* at L1 in control and *unc-30(191)* animals. Wild-type: n=45, *unc-30(e191)*: n=45.
(**C**) *madd-4* locus with UNC-30 ChIP-Seq tracks. UNC-30 site I (TAATCC) is mutated with CRISPR/Cas9 to GCGCGC in two alleles, *kas31* and *kas32*.
(**D**) Quantification of GABAergic MNs expressing *madd-4L(syb624 [2xN-LS::mScarlet::SL2::madd-4L])* at L4 in control (*syb624*) and *kas31* and *kas32* animals. n = 8. ns: not significant. For panels **B** and **D**, animals carry a cholinergic MN reporter (*otIs354 [cho-1(fosmid)::SL2::YFP::H2B]*). Box and whisker plots show median, lower, and upper quartiles – whiskers represent minimum and maximum. Black circles depict values. One-way ANOVA followed by Sidak's multiple comparison test. ns: not significant.

Development • Supplementary information

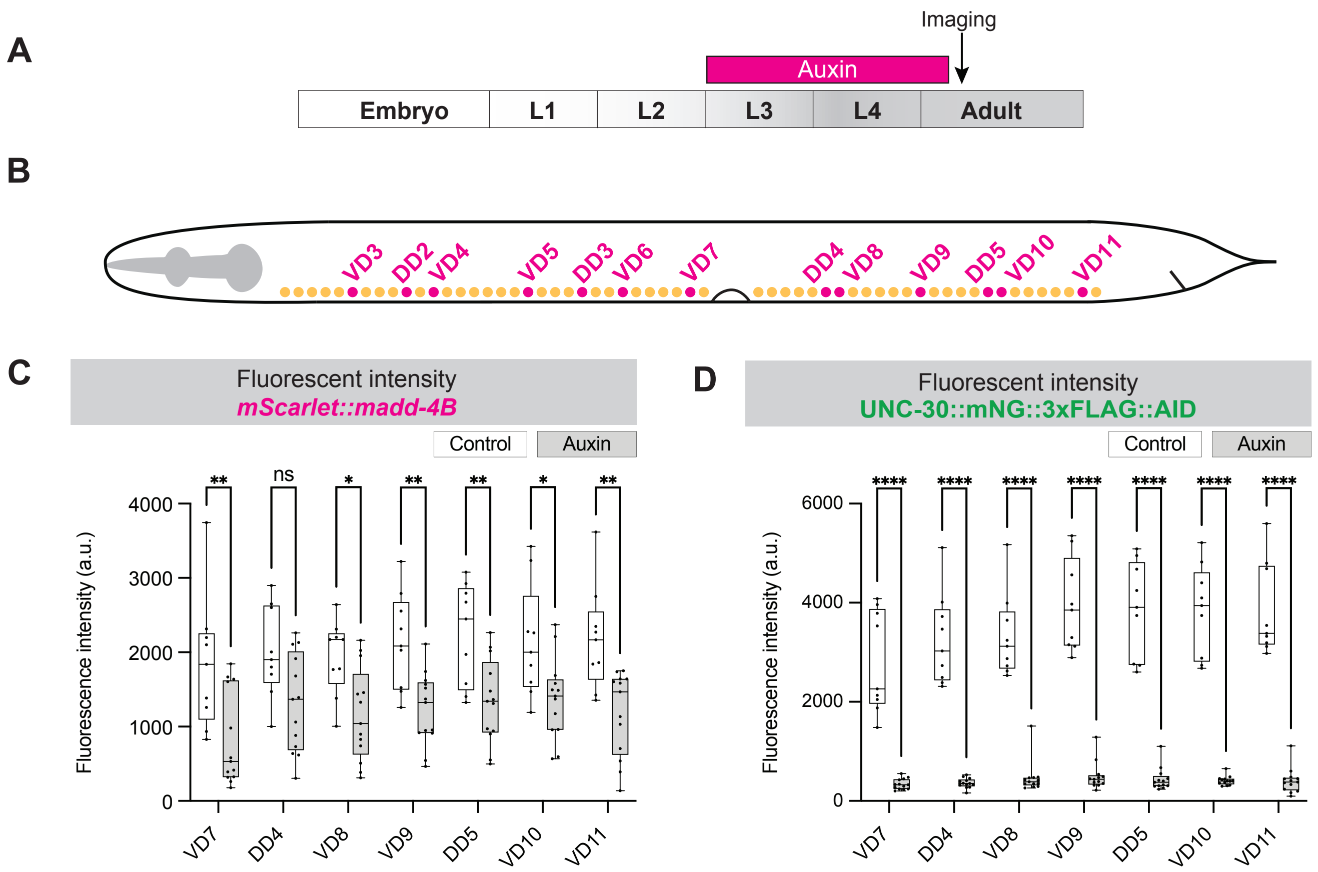

Fig. S6. UNC-30 is required to maintain *madd-4B* expression in GABAergic MNs. Related to Fig. 5.
(**A**) Schematic of auxin treatment timeline.
(**B**) Schematic of GABAergic (magenta) and cholinergic (yellow) nerve cord MNs.
(**C-D**) Quantification of *madd-4B (syb623[2xNLS::mScarlet::SL2::madd-4B])* or UNC-30(*syb2344* [UNC-30::mNG::3xFLAG::AID]) fluorescent intensity in individual posteri-orGABAergic MNs. Animals express TIR1 pan-somatically (*ieSi57 [Peft-3::TIR1::mRuby]*). Box and whisker plots show median, lower, and upper quartiles – whiskers represent mini-mum and maximum. Black circles depict values. Two-way ANOVA followed by Sidak's multiple comparison test. ****p<0.0001. Control: n=11, Auxin-treated=10.

Development • Supplementary information

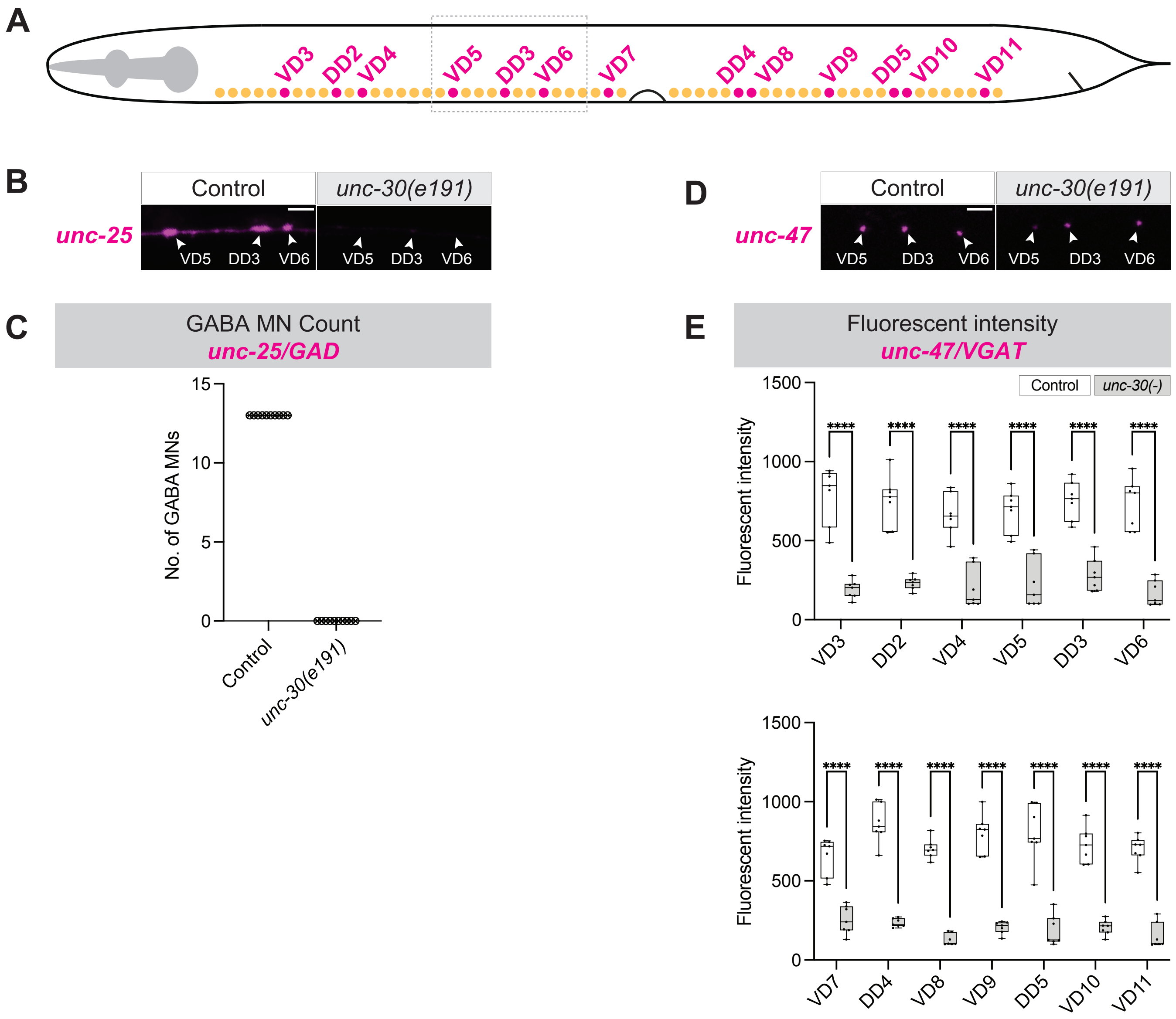

Fig. S7. UNC-30 activates *unc-25/GAD* and *unc-47/VGAT* expression in GABA motor neurons. Related to Fig. 8.

(**A**) GABAergic (magenta) and cholinergic (yellow) MNs. Dashed box depicts imaged area shown in (B, D).
(**B**) Fluorescence micrographs of *unc-25(hpIs88 [unc-25p::mCherry)* in control and *unc-30(191)* animals. White arrowheads: GABAergic MNs. Stage: L4.
(**C**) Quantification of the number of GABAergic nerve cord MNs cord expressing *unc-25 (hpIs88 [unc-25p::mCherry)*, as shown in (B). Box and whisker plots show median, lower, and upper quartiles – whiskers represent minimum and maximum. Black circles depict values. Control: n=10, *unc-30(e191)*: n=10.
(**D**) Fluorescence micrographs of *unc-47(otIs565 [unc-47(fosmid)::SL2::H2B::mChopti)* in control and *unc-30(191)* animals. White arrowheads: GABAergic MNs. Stage: L4.
(**E**) Quantification of *unc-47(otIs565 [unc-47(fosmid)::SL2::H2B::mChopti)* fluorescent intensity in GABAergic MNs. Two-way ANOVA followed by Sidak's multiple comparison test. ****p<0.0001. Black circles depict values. Control: n=8, *unc-30(e191)*: n=8.

Development • Supplementary information

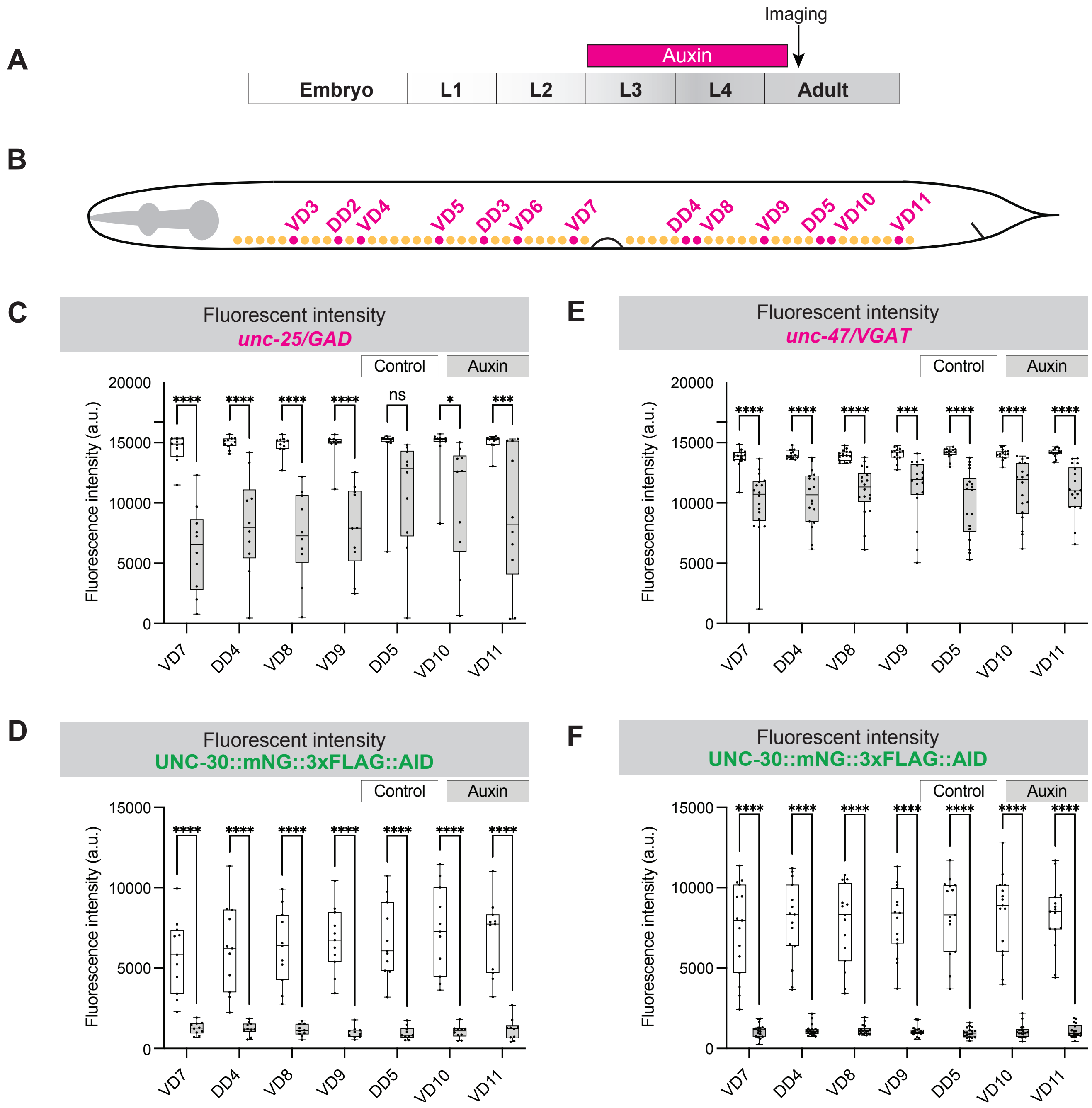

Fig. S8. UNC-30 is required to maintain expression of GABA biosynthesis genes in MNs. Related to Fig. 8.

(**A**) Schematic of auxin treatment timeline.

(**B**) Schematic of GABAergic (magenta) and cholinergic (yellow) MNs.

(**C-D**) Quantification of *unc-25 (hpIs88 [unc-25p::mCherry)* or UNC-30 (syb2344 [UNC-30::mNG::3xFLAG::AID]) fluorescent intensity in individual posterior GABAergic MNs. Animals express TIR1 pan-somatically (*ieSi57 [Peft-3::TIR1::mRuby]*). Control: n=11, Auxin-treat-ed=10.

(**E-F**) Quantification of *unc-47 (otIs565 [unc-47(fosmid)::SL2::H2B::mChopti)*) or UNC-30 (*syb2344* [UNC-30::mNG::3xFLAG::AID]) fluorescent intensity in posterior GABAergic MNs. Animals express TIR1 pan-somatically (*ieSi57 [Peft-3::TIR1::mRuby]*). Control: n=15, Auxin-treat-ed=18.Box and whisker plots show median, lower, and upper quartiles – whiskers represent minimum and maximum. Black circles depict values. Two-way ANOVA followed by Sidak's multi-ple comparison test. *p<0.05, ****p<0.0001. ns: not significant.

Development • Supplementary information

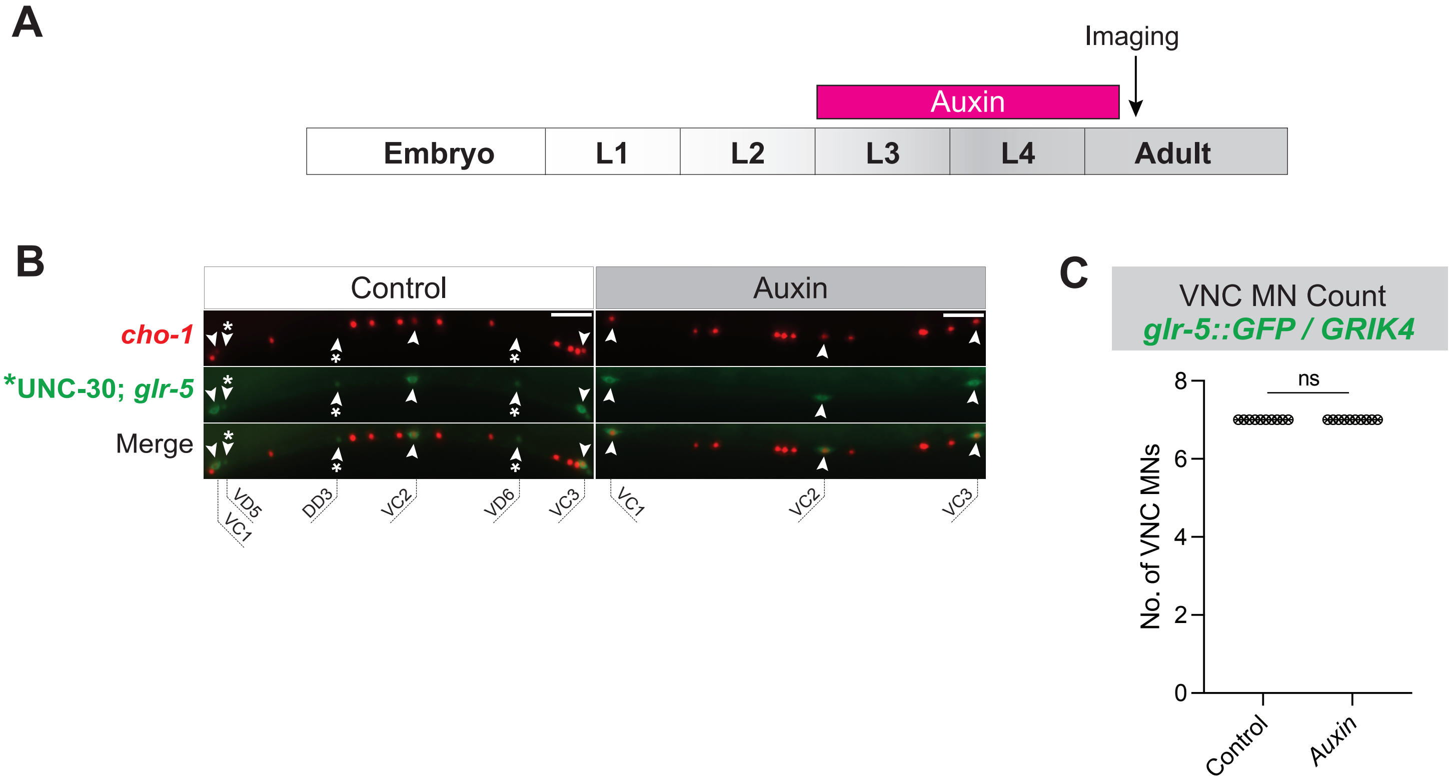

Fig. S9. UNC-30 is not required to maintain *glr-5/GRIK4* gene expression. Related to Fig. 9.
(**A**) Schematic of auxin treatment.
(**B**) Fluorescence micrographs of *icIs270 [glr-5::GFP],* UNC-30 (*syb2344*[UNC-30::mNG::3xFLAG::AID]), and a cholinergic MN reporter (*otIs544 [cho-1(fos-mid)::SL2::mCherry::H2B + pha-1(+)]*) in control (EtOH treated) and auxin-treated animals. White arrowheads: VC MNs. White arrowheads with asterisk: GABA MNs. scale bar: 10 μm.
(**C**) Quantification of MNs in the VNC expressing *icIs270 [glr-5::GFP]*, as shown in (f). Black circles depict values. Control: n=8, *unc-30(e191)*: n=8. ns: not significant.

Development • Supplementary information

Table S1. List of *C. elegans* strains used in this study.

Available for download at
https://journals.biologists.com/dev/article-lookup/doi/10.1242/dev.202733#supplementary-data

Table S2. List of primers used in this study.

Available for download at
https://journals.biologists.com/dev/article-lookup/doi/10.1242/dev.202733#supplementary-data

Development • Supplementary information
