# Supplementary material for: UNC-30/PITX coordinates neurotransmitter identity with postsynaptic GABA receptor clustering
Source: Development. 2024 Aug 27;151(16):dev202733. doi: 10.1242/dev.202733 (PMC11385328; doi:10.1242/dev.202733)
Supplement: Supplementary file 1 [file develop-151-202733_review_history.pdf]

UNC-30/PITX coordinates neurotransmitter identity with postsynaptic GABA receptor clustering

Edgar Correa, Morgane Mialon, Mélissa Cizeron, Jean-Louis Bessereau, Berangere Pinan-Lucarre and Paschalis Kratsios
DOI: 10.1242/dev.202733

Editor: François Guillemot

Review timeline
Original submission: 26 January 2024
Editorial decision: 26 February 2024
First revision received: 19 June 2024
Accepted: 10 July 2024

Original submission

First decision letter

MS ID#: DEVELOP/2024/202733

MS TITLE: UNC-30/PITX coordinates neurotransmitter identity with postsynaptic GABA receptor clustering

AUTHORS: Edgar Correa, Morgane Mialon, Melissa Cizeron, Jean-Louis Bessereau, Berangere Pinan-Lucarre, and Paschalis Kratsios

I have now received the reports of three referees on your manuscript, and I have reached a decision. The referees' comments are appended below, or you can access them online: please go to BenchPress and click on the 'Manuscripts with Decisions' queue in the Author Area.

As you will see, all the referees express great interest in your work, but they also have significant criticisms and recommend a substantial revision of your manuscript before we can consider publication. If you are able to revise the manuscript along the lines suggested, which may involve further experiments, I will be happy receive a revised version of the manuscript. Your revised paper will be re-reviewed by one or more of the original referees, and acceptance of your manuscript will depend on your addressing satisfactorily the reviewers' major concerns. Please also note that Development will normally permit only one round of major revision. If it would be helpful, you are welcome to contact us to discuss your revision in greater detail. Please send us a point-by-point response indicating your plans for addressing the referees' comments, and we will look over this and provide further guidance.

Please attend to all of the reviewers' comments and ensure that you clearly highlight all changes made in the revised manuscript. Please avoid using 'Tracked changes' in Word files as these are lost in PDF conversion. I should be grateful if you would also provide a point-by-point response detailing how you have dealt with the points raised by the reviewers in the 'Response to Reviewers' box. If you do not agree with any of their criticisms or suggestions please explain clearly why this is so.

Reviewer 1

Advance summary and potential significance to field

© 2024. Published by The Company of Biologists under the terms of the Creative Commons Attribution License (https://creativecommons.org/licenses/by/4.0/).

In this study, Correa and colleagues reveal that the expression of GABA synthesis pathway genes (UNC-25 and UNC-47) and a regulator of GABA receptor localization on post-synaptic muscle (MADD-4B) are coordinately regulated in the ventral nerve cord GABA neurons by the UNC-30/PITX terminal selector transcription factor. They further show that UNC-30 is continuously required during development to control GABA pathway gene expression and GABA neuron function. Additional work revealed that UNC-30 acts as both an activator (MADD-4B) and repressor (MADD-4L) to ensure faithful post-synaptic receptor expression and using ChIP-Seq data identified several other UNC-30 target genes in the GABA motor neurons.

The most interesting part of this study is the coordinated regulation of pre- and post-synaptic regulators by the same factor (UNC-30). Even though this coordinated role was previously shown by UNC-3 in the cholinergic neurons, the current study posits that such coordination may be a common phenomenon. Thus this is a significant finding for the neuroscience field. The data presented justify the conclusions drawn with one exception detailed below.

Comments for the author

Major points

On lines 219-220, the authors state that ‘we conclude that unc-30 controls GABAAR clustering in an indirect (non-cell autonomous) manner.’ However, the authors have not categorically shown that UNC-30 acts in the GABA motors neurons to control this, either through D-type neuron rescue experiments or D-type neuron-specific AID.

The authors did rescue madd-4B in the GABA motor neurons (Fig 2h) so the relevant promoter to perform this experiment is available.

Minor points

Line 64 - change flies to fruit flies (Drosophila melanogaster)

Line 72 - change fruit flies (Drosophila) to D. melanogaster

Line 196 - italicize -49 in unc-49

Lines 357 to 360 - Auxin-treated animals exhibited a mild reduction in the total number of mScarlet::madd-4B-expressing MNs (Figure 5f-g), but a significant reduction in mScarlet fluorescent intensity levels in all individual GABA MNs (Figure 5f-h, Supplementary Figure 4) - modify this sentence. The data both 5g and 5h are significant.

Line 729 - The madd-4(tr185) allele carries a mutation in exon 1 of madd-4B. Be more specific regarding the nature of the mutation.

Line 737 - Change wild-type to control to match the figure

Line 743 – Change (TAATCC sites I, II, II) II should be III

Line 760 - Change wild-type to control to match the figure. Change unc-30(e191: to unc-30(e191)

Line 785 – (e) Fluorescence micrographs of madd-L – missing the 4.

Line 821 - In panels e, h, and i: Box and whisker plots show median, lower, and upper quartiles whiskers represent minimum and maximum. Black circles depict values. g is missing?

Describe the unc-30(e191) consistently - e.g. line 205 = strong loss-of-function; line 367 = constitutive null

Describe transgene and CRISPR consistently – either unc-30::mNG::3xFLAG::AID allele (syb2344) or UNC-30::mNG::3xFLAG::AID allele (syb2344)

© 2024. Published by The Company of Biologists under the terms of the Creative Commons Attribution License (https://creativecommons.org/licenses/by/4.0/).

The specific promoter used for GABAp rescue of madd-4B is not defined or cited.
Also, this rescue strain is not present on the strain list.

Figure changes

Figure 6j needs to show the phenotype of each animal with dot points

Figure 5g – remove this graph since both 5h and 5i present a much stronger and clear phenotype.

Figure 3a - Schematic representation of ∆506bp, Figure 3a could be clearer by adding this allele number to the image.

Figure 3c-e, Figure 4b, Figure 5f Some arrowheads are solid other not. Figure legend does not mention this difference.

Figure 3b - Levels of expression of madd-4b in unc-30 mutants appear to be as strong as WT worms in Figure 3b for the VD8 neuron. This is inconsistent with the data display on the graph. A more representative image would help or is the arrowhead pointing to the wrong spot?

Be more consistent with nomenclature. madd-4S should be madd-4B in figure 4b.

Figure 4d,f The label above the graph states fluorescent intensity but the scoring was neuronal number and not intensity measurements.

Reviewer 2

Advance summary and potential significance to field

This interesting study from Correa and colleagues addresses important questions about the relationship between presynaptic differentiation and postsynaptic organization. Specifically, the study investigates the coordination of neurotransmitter identity and postsynaptic receptor clustering through the actions of the UNC-30/Pitx terminal selector transcription factor in C. elegans GABAergic motor neurons. UNC-30 is required for the expression of genes that indicate GABAergic identity (unc-25/GADD, unc-47/vesicular GABA transporter etc). The authors show here that UNC-30 also regulates the clustering of postsynaptic GABA A receptors through transcriptional control of the secreted synaptic organizer madd-4B, supporting a model for coordination between pre- and post-synaptic development through transcriptional mechanisms in the presynaptic neuron. These findings closely align with previous findings from the authors' labs supporting coordination of cholinergic identity and the clustering of postsynaptic receptors through the UNC-3 terminal selector. The present study extends this model for pre- and postsynaptic coordination by adding a number of additional interesting observations. The authors provide evidence that continuous expression of unc-30 is required for the maintenance of GABA identity genes. The authors also show that UNC-30 also represses the expression of another madd-4 isoform involved in cholinergic synapse development, suggesting previously unrecognized dual transcriptional repressor/activator roles for UNC-30. Finally, the authors suggest additional targets for direct transcriptional activation and repression by UNC-30. These are significant advances.
Overall, the manuscript is well-written, the experiments clearly performed and interpreted, and limitations acknowledged. I have just a few questions/recommendations.

Comments for the author

1. My main comment centers around the question of a continuous requirement for UNC-30 in the maintenance of GABAR clustering. The authors imply a requirement in lines 362-363 and later acknowledge this as a limitation of the study, but this seems straightforward to test. It seems an important experiment to validate the coordination between continuous presynaptic actions of the terminal selector and postsynaptic organization. For example, it is possible that madd-4B expression/secretion is required to initiate clustering but not strongly required for maintenance, as this may rely on other factors intrinsic to the postsynaptic cell.

© 2024. Published by The Company of Biologists under the terms of the Creative Commons Attribution License (https://creativecommons.org/licenses/by/4.0/).

2. Figure 2: The distribution of GABARs looks quite different when comparing across unc-30 and madd-4 mutants. Does this suggest a role for cholinergic madd-4L in regulating GABARs or perhaps implicate additional pathways regulated by unc-30? The authors should comment.

3. Figure 6: It seems expression of unc-25 is more strongly dependent on UNC-30 than expression of unc-47. Is this consistent with prior observations for unc-30 mutants or might this indicate a more stringent requirement for continuous expression in the case of unc-25?

Reviewer 3

Advance summary and potential significance to field

Correa et al. expand on previous findings from their groups on 1) the role of madd-4 (isoform B) as organizer of the postsynaptic density in the GABAergic neuro muscular junction (NMJ). MADD-4B acts non-cell autonomously: it is expressed and released from the presynaptic GABAergic motor neurons (MNs) to induce GABAR clustering and 2) the role of UNC-30 as terminal selector for GABAergic VD, DD MNs. In this work, authors now describe madd-4b as an additional UNC-30 direct target. Although it is well stablished that terminal selectors have broad actions in several neuronal features, including axon guidance or synaptic remodelling in temporal and sexual maturation, the specific direct targets with these actions are poorly characterized. This work provides a nice new example for this type of gene regulatory network. Additionally, Degron strategy is used to show that UNC-30 is continuously required to activate its targets, as has been previously shown for other terminal selectors in other neuronal contexts. Finally, they describe that UNC-30 acts directly as activator of madd-4b and repressor of madd-4l, authors also find additional targets of UNC-30 acting as repressor.
The manuscript is clear, data is solid, and analysis robust.
I think characterization of madd-4b allele compared to unc-30 mutant needs additional work, in addition it would be nice to assess if repressive actions of terminal selectors are also sustained throughout the life of the animal or only required to establish initial repression, which I don't think has been checked in any neuron type or system before.

Comments for the author

1. Figure 2:
Regarding unc-30 phenotype, it is not clear if, in addition to lack of correlation in pre-post synaptic GABA structures there are additional phenotypes:
a) Are there defects (either up or down) in unc-49 expression in muscle cells?
b) Is AchR distribution, in respect to GABA and ACh boutons affected in unc-30 mutants?
c) Do AchR overlap with GABAR in unc-30 mutants? Line 214, It is mentioned that GABAR localize opposite ACh presynapse (figure 2d) but there is no quantification of the phenotype.
d) Pearson correlation values are informative, but I would suggest to include fluorescence profiles similar to Pinan-Lucarre et al 2014 which are more illustrative of the phenotypes. Also, graphs indicate Pearson coefficient, but scales are 0 to 1. Are r or r2 values represented?

Regarding madd-4(tr185) phenotype and comparison to unc-30 mutants:
Figure 2 g-h: From pics comparing B and g, phenotypes seem different. In unc-30 mutants there seems to be an anti-correlation of GABA boutons with GABAR while in madd-4(tr185) seems there is an expansion of the GABAR domain and not so much an exclusion from GABA boutons. This perception from the picture is supported by the differences in pearson's correlation index between unc-30 mutant (aprox 0.2) and madd-4(tr185) (aprox. 0.5). IS this difference significant? Can authors analyse in more detail GABAR, AChR distribution and GABA boutons in both mutants? How is the correlation index in the double unc-30; madd4b mutants? Could the different phenotypes in the single mutants be explained by the expression of madd-4b in cholinergic motorneurons? (suggested experiment: does expression of madd-4B under a cholinergic prom in the double madd-4b/unc-30 mutant restores the anti-correlation index of unc-30 single mutant?) In addition, does madd-4B expression in cholinergic neurons rescue madd-4(tr185) phenotype?

© 2024. Published by The Company of Biologists under the terms of the Creative Commons Attribution License (https://creativecommons.org/licenses/by/4.0/).

2. Figure 2 needed control: for the tr185 allele, could authors please show by RT-qPCR that isoform B is affected and isoform 4L is not?

3. Is the phenotype of GABAR/GABA bouton correlation of madd-4(tr185) the same of the madd-4 null?

4. Figure 2g, similar to the question in unc-30 mutants, is unc-49 expression affected in madd-4(tr185) allele?

5. Figure 4: The repressive action of unc-30 is very interesting. Does point mutation of unc-30 binding site from the 2.9 kb prom lead to upregulation of GFP in GABA MN?

6. Is unc-30 action also continuously required to repress the targets (madd4l or glr-5, or unc-53)

7. Methods: it is mentioned that each experiment is done in two replicates, is the n in the figures representing values for both replicates together or values of one of the two replicates? Please clarify. Figure 2, different statistical methods are used in C and in h, but are the same kind of data. Could authors clarify?

8. Minor. Text:

- Abstract: "unc-30, the terminal selector of GABA MN identity". There are additional GABA MN not dependent on UNC-30, would be better to say "unc-30, the terminal selector of VD,DD GABA MN identity".
- line 55: in addition to NT switching maybe authors want to mention dual NTs in some neuron types?
- line 65: "neuron-type specific transcription factors, termed TS". What do you mean by neuron-type specific TFs?
- line 70: TS for 111 of 118 neuron types. There are TS for 117 of 118 (only RIM remains orphan)
- Line 79: there are several examples of NT maintenance in elegans using either temperature sensitive alleles, postembryonic RNAi or degron system.
- Line 386: is UNC-30 is also
- Line 387: unc-30 needs italics
- Line 579: would be nice to add a sentence or two on the findings for UNC-30 dual role in glia.
- Line 622, allele in italics
- Some figure panels could go to supplementary information, such as the quantification of UNC-30 levels upon auxin (Figure 5i, figure 6e, 6h) and summary panels in each figure are a bit repetitive. Could just maintain the one in figure 7.
- Figure 4 d and F, title of the graph says fluorescent intensity but the graph indicates number of neurons, not intensities.

First revision

Author response to reviewers' comments

RESPONSE TO REVIEWERS - DEVELOP/2024/202733

We thank all three reviewers for their time and constructive comments. We have addressed all comments via new experiments/analyses and text modifications.

Reviewer 1
Advance Summary and Potential Significance to Field:

In this study, Correa and colleagues reveal that the expression of GABA synthesis pathway genes (UNC-25 and UNC-47) and a regulator of GABA receptor localization on post-synaptic muscle (MADD-4B) are coordinately regulated in the ventral nerve cord GABA neurons by the UNC-30/PITX terminal selector transcription factor. They further show that UNC-30 is continuously required during

© 2024. Published by The Company of Biologists under the terms of the Creative Commons Attribution License (https://creativecommons.org/licenses/by/4.0/).

development to control GABA pathway gene expression and GABA neuron function. Additional work revealed that UNC-30 acts as both an activator (MADD- 4B) and repressor (MADD-4L) to ensure faithful post-synaptic receptor expression and using ChIP-Seq data identified several other UNC-30 target genes in the GABA motor neurons.

The most interesting part of this study is the coordinated regulation of pre- and post-synaptic regulators by the same factor (UNC-30). Even though this coordinated role was previously shown by UNC-3 in the cholinergic neurons, the current study posits that such coordination may be a common phenomenon. Thus, this is a significant finding for the neuroscience field. The data presented justify the conclusions drawn with one exception detailed below.

Reviewer 1 Comments for the Author:

-
Major points

On lines 219-220, the authors state that 'we conclude that unc-30 controls GABAAR clustering in an indirect (non-cell autonomous) manner.' However, the authors have not categorically shown that UNC-30 acts in the GABA motors neurons to control this, either through D-type neuron rescue experiments or D-type neuron-specific AID.

The authors did rescue madd-4B in the GABA motor neurons (Fig 2h) so the relevant promoter to perform this experiment is available.

We agree that rescue experiments are of value. The promoter we used for *madd-4B* rescue is *unc-47* **Figure 4** (old Fig. 2), which is a target gene of UNC-30. The expression of an *unc-47* transgenic reporter (that contains the *unc-47* prom sequence) is dramatically reduced/eliminated in *unc-30 (e191)* animals (PMID: 10414952), and this is also the case for other known UNC-30 targets (e.g., *unc-25*, *flp-13*, *acr-14*) (PMID: 15723795). Consistently, we also observed strong effects on *unc-25* and *unc-47* expression in *unc-30 (e191)* animals (new **Suppl. Fig. 8**). Hence, we unfortunately do not have an *unc-30*-independent promoter to conduct rescue.

To address the reviewer's concern:

a) We note in **Results** that previous studies employing antibody staining showed that *unc-30* expression is remarkably specific and restricted to GABA MNs and a few additional neurons in the head. The literature reports no expression in muscle cells, on which we observe GABAR clustering defects.
b) In **Supplementary Figures 2** and **3**, we analyze available scRNA-Seq data during embryogenesis. In the MS lineage, we only observe *unc-30* transcripts in GLR glia cells, and not in muscle cells (**Suppl. Fig. 2**).
c) In the AB lineage that gives rise to neurons, the expression of *unc-30* is remarkably specific and restricted in the nervous system. In addition to GABA motor neurons (DD, VD), only 10 other neuron types (PVP, RIC, RIB, PVP, AIB, AVJ, AVH, RIA, AVD, ASG) of the total 118 in *C. elegans* express *unc-30* (**Suppl. Fig. 3**).

We have toned down our conclusions in Results as follows: *"Because unc-30 is present in GABAergic MNs but not expressed in body wall muscles or muscle progenitor cells (**Supplementary Figures 2-3**)[20], it is likely that unc-30 controls GABAAR clustering in an indirect (non-cell autonomous) manner.*

Minor points

Line 64 - change flies to fruit flies (Drosophila melanogaster)

Done. Thanks.

© 2024. Published by The Company of Biologists under the terms of the Creative Commons Attribution License (https://creativecommons.org/licenses/by/4.0/).

Line 72 - change fruit flies (Drosophila) to D. melanogaster
Done.

Line 196 - italicize -49 in unc-49 Done.

Lines 357 to 360 - Auxin-treated animals exhibited a mild reduction in the total number of mScarlet::madd-4B-expressing MNs (Figure 5f-g), but a significant reduction in mScarlet fluorescent intensity levels in all individual GABA MNs (Figure 5f-h, Supplementary Figure 4) - modify this sentence. The data both 5g and 5h are significant.
Done.

Line 729 - The madd-4(tr185) allele carries a mutation in exon 1 of madd-4B. Be more specific regarding the nature of the mutation. ATG to ATA? Isoleucine.

We have updated the figure legend and figure accordingly.
The *tr185* mutation consists in a initiator methionine codon (ATG) switch to an isoleucine (ATA) Originally described in a previous study (PMID: 22014523), this mutation affects nucleotide #3 in the first codon of *madd-4B* sequence.

Line 737 - Change wild-type to control to match the figure
Done.

Line 743 – Change (TAATCC sites I, II, II) II should be III
Done.

Line 760 - Change wild-type to control to match the figure. Change unc-30(e191: to unc-30(e191)
Done.

Line 785 – (e) Fluorescence micrographs of madd-L – missing the 4.
Done.

Line 821 - In panels e, h, and i: Box and whisker plots show median, lower, and upper quartiles whiskers represent minimum and maximum. Black circles depict values. g is missing?
Done.

Describe the unc-30(e191) consistently - e.g. line 205 = strong loss-of-function; line 367 = constitutive null
For consistency, we now refer to *unc-30(e191)* as a strong LOF allele.

In Fig. 5a legend, we must improve the e191 allele.
Done.

Describe transgene and CRISPR consistently – either unc-30::mNG::3xFLAG::AID allele (syb2344) or UNC-30::mNG::3xFLAG::AID allele (syb2344)

In the manuscript, we follow standard *C. elegans* gene and protein nomenclature. The *unc-30* alleles are shown in italics and small letters: *unc-30 (e191)* or *unc- 30::mNG::3xFLAG::AID.*
The UNC-30 protein is shown in capital letters: UNC-30::mNG::3xFLAG::AID.

The specific promoter used for GABAp rescue of madd-4B is not defined or

© 2024. Published by The Company of Biologists under the terms of the Creative Commons Attribution License (https://creativecommons.org/licenses/by/4.0/).

cited. Also, this rescue strain is not present on the strain list.
Apologies for this oversight. This info is now included in the figure legend and stain list (**Suppl. File 1**).

Figure changes
Figure 6j needs to show the phenotype of each animal with dot points
Done. Thanks.

Figure 5g – remove this graph since both 5h and 5i present a much stronger and clear phenotype.
We agree and have removed panel 5g.

Figure 3a - Schematic representation of Δ506bp, Figure 3a could be clearer by adding this allele number to the image.
Done. Thanks.

Figure 3c-e, Figure 4b, Figure 5f Some arrowheads are solid other not. Figure legend does not mention this difference.
To avoid confusion, we now show only solid arrowheads to indicate GABAergic motor neurons.

Figure 3b - Levels of expression of madd-4b in unc-30 mutants appear to be as strong as WT worms in Figure 3b for the VD8 neuron. This is inconsistent with the data display on the graph. A more representative image would help or is the arrowhead pointing to the wrong spot?

We have replaced it with a more representative image. All the images in Figure 5b (old Figure 3b) were taken using the same microcopy settings/parameters, and all the quantifications were done based on those images

Be more consistent with nomenclature. madd-4S should be madd-4B in figure 4b. Change in Illustrator figure 4b to write madd-4B next to image.
We have checked the entire manuscript and figure panels. We now use only *madd-4B* for consistency.

Figure 4d,f The label above the graph states fluorescent intensity but the scoring was neuronal number and not intensity measurements.
We corrected this now. Thanks.

*****

<u>Reviewer 2</u>
Advance Summary and Potential Significance to Field:

This interesting study from Correa and colleagues addresses important questions about the relationship between presynaptic differentiation and postsynaptic organization. Specifically, the study investigates the coordination of neurotransmitter identity and postsynaptic receptor clustering through the actions of the UNC-30/Pitx terminal selector transcription factor in C. elegans GABAergic motor neurons. UNC-30 is required for the expression of genes that indicate GABAergic identity (unc- 25/GADD, unc-47/vesicular GABA transporter etc). The authors show here that UNC-30 also regulates the clustering of postsynaptic GABA A receptors through transcriptional control of the secreted synaptic organizer madd-4B, supporting a model for coordination between pre- and post- synaptic development through transcriptional mechanisms in the presynaptic neuron. These findings closely align with previous findings from the authors' labs supporting coordination of cholinergic identity and the clustering of postsynaptic receptors through the UNC-3 terminal selector. The present study extends this model for pre- and postsynaptic coordination by adding a number of additional interesting observations. The authors provide evidence that continuous expression of unc-30 is required for the maintenance of GABA

© 2024. Published by The Company of Biologists under the terms of the Creative Commons Attribution License (https://creativecommons.org/licenses/by/4.0/).

identity genes. The authors also show that UNC-30 also represses the expression of another madd-4 isoform involved in cholinergic synapse development, suggesting previously unrecognized dual transcriptional repressor/activator roles for UNC-30. Finally, the authors suggest additional targets for direct transcriptional activation and repression by UNC-30. These are significant advances. Overall, the manuscript is well-written, the experiments clearly performed and interpreted, and limitations acknowledged. I have just a few questions/recommendations.

Reviewer 2 Comments for the Author:

1. My main comment centers around the question of a continuous requirement for UNC-30 in the maintenance of GABAR clustering. The authors imply a requirement in lines 362-363 and later acknowledge this as a limitation of the study, but this seems straightforward to test. It seems an important experiment to validate the coordination between continuous presynaptic actions of the terminal selector and postsynaptic organization. For example, it is possible that madd-4B expression/secretion is required to initiate clustering, but not strongly required for maintenance, as this may rely on other factors intrinsic to the postsynaptic cell.

We tried to address this very important question. For the AID experiments to assess a continuous requirement for UNC-30 on *madd-4B* (**Figure 7**), we used the *ieSi57 [eft-3prom::TIR1::mRuby]* pansomatic TIR1 transgene. To test for a continuous UNC-30 requirement on GABAR clustering, we had to switch to a different TIR1 line because the ubiquitous mRuby signal [red fluorescence] from the *ieSi57 [eft-3prom::TIR1::mRuby]* TIR1 transgene masks the dim red signal from UNC-49::RFP (GABAR marker). Regrettably, the alternate TIR1 line we used did not achieve UNC-30 protein depletion upon auxin treatment.

To address the reviewer's concern, we added the following sentence in **Discussion**:

"Alternatively, it is possible that MADD-4B secretion is only required to initiate GABAAR clustering, but subsequent maintenance of such clustering may rely on other factors intrinsic to the postsynaptic cell."

2. Figure 2: The distribution of GABARs looks quite different when comparing across unc-30 and madd-4 mutants. Does this suggest a role for cholinergic madd-4L in regulating GABARs or perhaps implicate additional pathways regulated by unc-30? The authors should comment.

Our purpose here was to highlight a similarity of phenotypes between the *unc-30* and *madd-4(tr185)* mutants: ectopic clustering of GABAAR at cholinergic NMJs. We have now expanded our analysis of GABAergic and cholinergic synapses in the *unc-30* mutant to strengthen this concept. New **Figures 2 and 3** and new **Suppl. Fig. 1** quantify the distribution and correlation of GABA and ACh boutons, as well as GABARs and AChRs in *unc-30(e191)* mutants. New **Figure 4** provides a correlation analysis between GABA boutons and GABARs in *madd-4B(tr185)* mutants with and without GABA neuron- specific rescue.

However, we also underscore a different distribution of GABARs between *unc-30(e191)* and *madd-4B(tr185)* mutants. Indeed, GABAARs partially cluster at GABAergic neuromuscular junctions in the *madd-4(tr185)* mutant while they fail to properly cluster at these synapses in the *unc-30(e191)* mutant. The different distribution of GABARs is likely due to the following reasons:

- Additional yet-to-be-identified UNC-30 target genes may also act as GABA synapse organizers. It is possible that – in addition to madd-4B - their expression is also affected in *unc-30(e191)* mutants, resulting in a different GABAR distribution phenotype between *unc- 30(e191)* and *madd-4B(tr185)* mutants.
- In the *madd-4(tr185)* mutant, these additional genes might operate in parallel to MADD-4L, which has been shown to trap GABAARs at cholinergic neuromuscular junctions. These separate mechanisms might explain the localization of GABAARs at both cholinergic and GABAergic NMJs.

We now clearly mention these two possibilities in **Limitations**.

© 2024. Published by The Company of Biologists under the terms of the Creative Commons Attribution License (https://creativecommons.org/licenses/by/4.0/).

3. Figure 6: It seems expression of unc-25 is more strongly dependent on UNC-30 than expression of unc-47. Is this consistent with prior observations for unc-30 mutants or might this indicate a more stringent requirement for continuous expression in the case of unc-25?

To address this, we assessed *unc-25* and *unc-47* reporter gene expression in *unc-30(e191)* null mutants (new **Suppl. Fig 8**). The null mutant results mirror the auxin data, i.e., *unc-25* expression depends more strongly on *unc-30* gene activity than *unc-47* expression. However, we prefer to refrain from making strong conclusions on differential dependence of *unc-25* and *unc-47*. The reason is that different reagents were used for each gene: *unc-25::RFP* is a promoter fusion that carries a *cis-* regulatory element of 1.8 kb to drive RFP expression, whereas the *unc-47* reporter is a fosmid-based reporter, i.e., it is a ~30kb genomic clone that contains the *unc-47* gene locus and multiple other genes. From past experience with other transcription factor targets, we know that when we compare effects for the SAME target gene with a promoter fusion and a fosmid-based reporter, the effects tend to be stronger with a promoter fusion, likely due to the presence of the entire *cis*-regulatory landscape in fosmid-based reporters that allow for other factors to compensate.

*****

Reviewer 3
Advance Summary and Potential Significance to Field:

Correa et al. expand on previous findings from their groups on 1) the role of madd-4 (isoform B) as organizer of the postsynaptic density in the GABAergic neuro muscular junction (NMJ). MADD-4B acts non-cell autonomously: it is expressed and released from the presynaptic GABAergic motor neurons (MNs) to induce GABAR clustering and 2) the role of UNC-30 as terminal selector for GABAergic VD, DD MNs. In this work, authors now describe madd-4b as an additional UNC-30 direct target. Although it is well stablished that terminal selectors have broad actions in several neuronal features, including axon guidance or synaptic remodelling in temporal and sexual maturation, the specific direct targets with these actions are poorly characterized. This work provides a nice new example for this type of gene regulatory network. Additionally, Degron strategy is used to show that UNC-30 is continuously required to activate its targets, as has been previously shown for other terminal selectors in other neuronal contexts. Finally, they describe that UNC-30 acts directly as activator of madd-4b and repressor of madd-4l, authors also find additional targets of UNC-30 acting as repressor.
The manuscript is clear, data is solid, and analysis robust. I think characterization of madd-4b allele compared to unc-30 mutant needs additional work, in addition it would be nice to assess if repressive actions of terminal selectors are also sustained throughout the life of the animal or only required to establish initial repression, which I don't think has been checked in any neuron type or system before.

Reviewer 3 Comments for the Author:

1. Figure 2:
Regarding unc-30 phenotype, it is not clear if, in addition to lack of correlation in pre-post synaptic GABA structures there are additional phenotypes:
a) Are there defects (either up or down) in unc-49 expression in muscle cells?

Motivated by these comments, we expanded the synaptic analysis of *unc-30* mutants. In the revised **Figures 2D, 2H, 3B**, and **3F**, we quantify the levels of expression of an endogenous UNC-49::RFP reporter in muscle cells, using UNC-49::RFP red fluorescence as a proxy. We did not observe any differences between control and *unc-30(e191)* animals.
We also observed no differences in UNC-49::RFP red fluorescence levels in the muscle cells of control and *madd-4B(tr185)* animals (new **Figure 4E**).
Therefore, these data emphasize that the *unc-30(e191)* and *madd-4(tr185)* mutants display strong defects in the localization of GABAARs, and not in the levels of expression of GABAARs.

© 2024. Published by The Company of Biologists under the terms of the Creative Commons Attribution License (https://creativecommons.org/licenses/by/4.0/).

b) Is AchR distribution, in respect to GABA and ACh boutons affected in unc-30 mutants?

Pearson's correlation coefficient between an endogenous AChR marker (UNC-29-tagRFP) and an ACh bouton marker (CLA-1-BFP) did not show any differences between control and *unc-30(e191)* mutants (**Suppl. Fig. 1F**), indicating that the distribution of AChRs is normal in *unc-30(e191)* animals.

In new **Suppl. Fig. 1E**, we also quantify the levels of expression of the endogenous AChR reporter (UNC-29::tagRFP) in muscle cells, using red fluorescence as a proxy. We did not observe any differences between control and *unc-30(e191)* animals. Similarly, we now show that the density of ACh boutons (assessed via the active zone marker CLA-1) is not affected in *unc-30(e191)* animals at the ventral (**Fig. 3D**) or and dorsal (**Fig. 3H**) nerve cords (**Suppl. Fig. 1G**).

c) Do AchR overlap with GABAR in unc-30 mutants? Line 214, It is mentioned that GABAR localize opposite ACh presynapse (figure 2d) but there is no quantification of the phenotype.

We have now quantified these effects. In revised **Figure 2**, we show that the correlation of GABA boutons and GABARs is lower in *unc-30(e191)* animals (**Fig. 2C, 2G**). On the other hand, the correlation between ACh boutons and GABARs is higher in *unc-30(e191)* animals compared to controls (**Fig. 3C, 3G**). These data support a key conclusion of our study: there is a loss of GABAR clustering across GABA boutons and a simultaneous gain of GABAR clustering across ACh boutons.

d) Pearson correlation values are informative, but I would suggest to include fluorescence profiles similar to Pinan-Lucarre et al 2014 which are more illustrative of the phenotypes. Also, graphs indicate Pearson coefficient, but scales are 0 to 1. Are r or r2 values represented?

We agree and have now included fluorescence profiles in **Figures 2, 3, 4,** and **Suppl. Fig. 1**. The bona fide Pearson's r values are shown, not the r^2. We added a sentence in the Methods as follows: *"Pearson's r values are indicated."*.

Regarding madd-4(tr185) phenotype and comparison to unc-30 mutants:
Figure 2 g-h: From pics comparing B and g, phenotypes seem different. In unc-30 mutants there seems to be an anti-correlation of GABA boutons with GABAR while in madd-4(tr185) seems there is an expansion of the GABAR domain and not so much an exclusion from GABA boutons. This perception from the picture is supported by the differences in pearson's correlation index between unc-30 mutant (aprox 0.2) and madd-4(tr185) (aprox. 0.5). Is this difference significant?

We cannot directly address whether the Pearson's values are significantly different between the *madd-4(tr185)* and the *unc-30(e191)* mutants because we used different presynaptic GABA markers in different sets of experiments. In *madd-4 (tr185)* mutants, we labeled GABA boutons with a transgenic marker (*krIs67[unc-47prom::snb-1::bfp]*. However, this unc-47 promoter relies on UNC-30 for activation in GABA motor neurons. Hence, to label GABA boutons in *unc-30(e191)* mutants, we developed a split GFP approach (*bab462 [cla-1::GFP11x7]; krSi342 [unc-30prom::gfp1-10]*).

Motivated by the reviewer's comment, we significantly expanded the synaptic analysis of both between *unc-30(e191)* and *madd-4B(tr185)* mutants. New **Figures 2** and **3**, and new **Suppl. Fig. 1** show quantifications and correlations of GABA and ACh bouton distribution, as well as GABAR localization in *unc-30 (e191)* mutants. New **Figure 4** provides a correlation analysis between GABA boutons and GABARs in *madd-4B(tr185)* mutants with and without GABA neuron-specific madd-4B rescue.

We agree with the reviewer and summarize here the similarities and differences of the synaptic phenotypes:

***unc-30* synaptic phenotype**: we show that the correlation of GABA boutons and GABARs is lower in *unc-30(e191)* animals (**Fig. 2C, 2G, Suppl. Fig. 1B**). On the other hand, the correlation between ACh boutons and GABARs is higher in *unc-30(e191)* animals compared to controls (**Fig. 3C, 3G**). These data support a key conclusion of our study: there is a loss of GABAR clustering across GABA boutons and a simultaneous gain of GABAR clustering across ACh boutons in *unc-30*

© 2024. Published by The Company of Biologists under the terms of the Creative Commons Attribution License (https://creativecommons.org/licenses/by/4.0/).

mutants.

***madd-4B(tr185)* synaptic phenotype**: we show that the correlation of GABA boutons and GABARs is lower in *madd-4B(tr185)* animals (**Fig. 4B**). In a previous study (PMID: 24896188), we have conducted a detailed analysis of the *madd-4B(tr185)* synaptic phenotype and showed that GABAAR clusters are not restricted to sites opposite of GABA boutons and rather face both GABAergic and cholinergic boutons.

In **Limitations,** we added the following text:
*"Further, although GABAARs undergo similar ectopic clustering at cholinergic neuromuscular junctions in both madd-4(tr185) and unc-30(e191) animals, we also note that there are differences in the GABAAR clustering defects observed. Indeed, GABAARs partially cluster at GABAergic neuromuscular junctions in the madd-4(tr185) mutant while they fail to properly cluster at these synapses in the unc- 30(e191) mutant (**Figure 2, 4**). This suggests that, in addition to madd-4B, other yet-to-be identified UNC-30 target genes may also act as GABA synapse organizers. In the madd-4(tr185) mutant, these additional genes may operate in parallel to MADD-4L, which has been shown to trap GABAARs at cholinergic neuromuscular junctions."*

Can authors analyze in more detail GABAR, AChR distribution and GABA boutons in both mutants? How is the correlation index in the double unc-30; madd4b mutants?

We have analyzed the *madd-4B(tr185)* synaptic phenotypes in previous studies (PMID: 24896188, 26028575). Consistent with these studies, we observe a lower correlation between GABAR clustering and GABA boutons (**Fig. 4D**). Our data expand further these previous studies by conducting a GABA motor neuron-specific rescue experiment: when we supply MADD-4B in these neurons, we correct the GABAR clustering defect on the muscle cells (**Fig. 4D**), indicating that MADD-4B is produced in - and secreted from - GABA motor neurons to control GABAR clustering.

As requested, we provide new data on GABAR, AChR, and GABA bouton localization in *unc-30 (e191)* mutans (**Fig. 2, 3, Suppl. Fig. 1**).

Could the different phenotypes in the single mutants be explained by the expression of madd-4b in cholinergic motorneurons? (suggested experiment: does expression of madd-4B under a cholinergic prom in the double madd-4b/unc-30 mutant restores the anti-correlation index of unc-30 single mutant?).
In addition, does madd-4B expression in cholinergic neurons rescue madd-4(tr185) phenotype?

Our main purpose here was to show a similarity of phenotypes between the *unc-30* and *madd-4(tr185)* mutants: ectopic clustering of GABAAR at cholinergic NMJs. We share the frustration of the reviewer for not understanding fully the role of specific *madd-4* isoforms left in the *unc-30* mutant.
However, analyzing the role of specific *madd-4* isoforms is a very challenging issue which has been investigated for seven years now in our lab, especially since MADD-4B and MADD-4L are further cleaved into smaller fragments, each showing specific synapse organizing properties (M. Cizeron, unpublished data). Given the complexity of these data, we feel that analyzing the role of specific *madd-4* isoforms in the *unc-30* mutant is beyond the scope of this manuscript. Eventually, since our data point to a complete rescue of GABAR distribution when *madd-4B* is expressed in the GABAergic motoneurons of the *madd-4(tr185)* mutant (**Fig. 4**), we did not express *madd-4B* in the cholinergic motoneurons.

In addition, we clarified that the different distribution of GABARs between *unc-30(e191)* and *madd- 4B(tr185)* mutants is likely due to the following reasons:

- Additional yet-to-be-identified UNC-30 target genes may also act as GABA synapse organizers. It is possible that - in addition to madd-4B - their expression is also affected in *unc-30(e191)* mutants, resulting in a different GABAR distribution phenotype between *unc- 30(e191)* and *madd-4B(tr185)* mutants.
- In the *madd-4(tr185)* mutant, these additional genes might operate in parallel to MADD-

© 2024. Published by The Company of Biologists under the terms of the Creative Commons Attribution License (https://creativecommons.org/licenses/by/4.0/).

4L, which have been shown to trap GABAARs at cholinergic neuromuscular junctions. These different mechanisms might explain the localization of GABAARs at both cholinergic and GABAergic NMJs.

In the revised manuscript, we account for both of these possibilities in **Limitations**.

2. Figure 2 needed control: for the tr185 allele, could authors please show by RT-qPCR that isoform B is affected and isoform 4L is not?

In a previous study (PMID: 24896188), we conducted a detailed analysis of the *madd-4B(tr185)* synaptic phenotype and important control experiments are included there. Notably, we have shown that MADD-4L clusters AChRs at excitatory neuromuscular junctions, including in the context of the *madd-4(tr185)* mutant. In addition, electrophysiological recordings showed wild-type AChR currents at the excitatory neuromuscular junction in the *madd-4(tr185)* mutant. These experiments indicate wild- type *madd-4L* expression and function in the *madd-4(tr185)* mutant.

3. Is the phenotype of GABAR/GABA bouton correlation of madd-4(tr185) the same of the madd-4 null?

We have previously conducted a detailed comparison between the *madd-4* null and *madd-4B(tr185)* synaptic phenotypes (PMID: 24896188, 26028575).
- In the *madd-4(tr185)* mutant, GABAAR clusters are not restricted to sites opposite of GABA boutons and rather face both GABAergic and cholinergic boutons.
- In the *madd-4* null mutant, some of the GABAAR diffuse away the nerve cords, in the muscle arms (PMID 24896188), while a limited amount of GABAAR clusters remain at the cords.

The different distribution of GABAARs these two mutants suggests that, in the *madd-4(tr185)* mutant, MADD-4L traps some part of the GABAAR pool at cholinergic neuromuscular junctions.

4. Figure 2g, similar to the question in unc-30 mutants, is unc-49 expression affected in madd-4(tr185) allele?

In new **Figure 4E**, we quantify the levels of expression of an endogenous UNC-49::RFP reporter in muscle cells, using UNC-49::RFP red fluorescence as a proxy. We did not observe any differences between control and *madd-4B(tr185)* animals, consistently with our previous study (PMID: 26028575). This latter study also showed wild-type currents elicited by pressure-ejection of GABA on muscle cells, indicating wild-type expression and function of GABAAR in the *madd-4B(tr185)* mutant.

5. Figure 4: The repressive action of unc-30 is very interesting. Does point mutation of unc-30 binding site from the 2.9 kb prom lead to upregulation of GFP in GABA MN?

ChIP-Seq and genetic data suggest that UNC-30 directly represses *madd-4L* (new **Figure 6**). Using CRISPR/Cas9, we mutated the canonical TAATCC UNC-30 binding site upstream of *madd-4L*. However, we observed no effect on the number of MNs expressing *mScarlet::madd-4L* **(Suppl. Fig. 5c-d**). We therefore conclude that UNC-30 indirectly represses *madd-4L* transcription. We revised the text to:

*"UNC-30 binding (peak I) upstream of madd-4L suggests UNC-30 directly represses madd-4L in GABAergic MNs. However, mutating the canonical UNC-30 binding sequence (WT site I: TAATCC site; MUT site I: GCGCGC) with CRISPR/Ca9 gene editing had no effect on the number of GABA MNs expressing mScarlet::madd-4L (**Supplementary Figure 5c-d**). We conclude that, in GABA MNs, UNC-30/PITX controls two isoforms of the same synapse organizer in opposite ways; it directly activates madd-4B and indirectly represses madd-4L (**Figure 6g**)"*

6. Is unc-30 action also continuously required to repress the targets (madd4l or glr-5, or unc-53)?

For various technical reasons (e.g., the mRuby signal [red fluorescence] from the *ieSi57 [eft-3prom::TIR1::mRuby]* TIR1 transgene masks the dim red signal from *mScarlet::madd-4L*), we conducted this experiment only for *glr-5::gfp*.

© 2024. Published by The Company of Biologists under the terms of the Creative Commons Attribution License (https://creativecommons.org/licenses/by/4.0/).

Despite robust AID-mediated UNC-30 depletion, we did not observe any effects on *glr-5::gfp* expression (**Suppl. Fig. 9**), suggesting that at late larval stages (L3-L4) UNC-30 is not required to repress *glr-5* in GABA motor neurons. We updated the **Results** accordingly.

7. Methods: it is mentioned that each experiment is done in two replicates, is the n in the figures representing values for both replicates together or values of one of the two replicates? Please clarify.

N in figures represents pooled data for each genotype, i.e., values for both replicates.

Figure 2, different statistical methods are used in C and in h, but are the same kind of data. Could authors clarify?

The synaptic data are shown in **Figures 2-4** and **Suppl. Fig. 1**
In **Figure 2**, we used a non-parametric Mann-Whitney test.
In **Figure 3** and **Suppl. Fig. 1**, we used Mann-Whitney test and Student's *t*-test. In **Figure 4**, we used Kruskal-Wallis followed by Dunn's post-test.
We added a comment in the Methods to explain the choice of statistical tests as follows: "*For experiments presented in Figures 2-4 and Supplementary Fig. 1, box and whisker plots show median, lower, and upper quartiles, and whiskers represent standard deviation (SD). For Pearson's correlation coefficient, fluorescence intensity and boutons density quantifications, Mann-Whitney tests (comparison of two genotypes) and Kruskal-Wallis followed by Dunn's post-tests (comparison of more than two genotypes) were performed if data did not follow a normal distribution (Shapiro normality test) or did not show equality of variances (Bartlett test). If data were normal and showed equality of variances, Student's t-tests were performed.*"

8. Minor. Text:

•Abstract: "unc-30, the terminal selector of GABA MN identity". There are additional GABA MN not dependent on UNC-30, would be better to say "unc-30, the terminal selector of VD,DD GABA MN identity".
We changed to: "*Here, we report that UNC-30 (PITX2/3), the terminal selector of GABA nerve cord motor neurons in C. elegans,..*"

•line 55: in addition to NT switching maybe authors want to mention dual NTs in some neuron types?
Done. Thanks.

•line 65: "neuron-type specific transcription factors, termed TS". What do you mean by neuron-type specific TFs?
We revised to "*transcription factors expressed in specific neuron types….*".

•line 70: TS for 111 of 118 neuron types. There are TS for 117 of 118 (only RIM remains orphan)
Done. There are predicted TS for 117 of 118 neuron types.

•Line 79: there are several examples of NT maintenance in elegans using either temperature sensitive alleles, postembryonic RNAi or degron system.
We agree. Out of the 117 predicted terminal selectors, a few of them (e.g., UNC-3, LIN-39, CHE-1, AST-1, UNC-86, CTBP-1), but no more than 10, have been tested for NT maintenance. It is therefore reasonable to state that "… their involvement in maintaining NT identity in later life stages remains poorly examined".

•Line 386: is UNC-30 is also
Fixed. Thanks.

•Line 387: unc-30 needs italics
Fixed. Thanks

© 2024. Published by The Company of Biologists under the terms of the Creative Commons Attribution License (https://creativecommons.org/licenses/by/4.0/).

•Line 579: would be nice to add a sentence or two on the findings for UNC-30 dual role in glia.
Done, we added the following: “*Intriguingly, a similar dual role for UNC-30 has recently been described in C. elegans glia, where it promotes GLR glia morphology and represses alternative mesodermal fates*[84].”

•Line 622, allele in
italics
Done. Thanks.

•Some figure panels could go to supplementary information, such as the quantification of UNC-30 levels upon auxin (Figure 5i, figure 6e, 6h) and summary panels in each figure are a bit repetitive. Could just maintain the one in figure 7.
Thank you for this suggestion. Our figures/data have been significantly re-organized.
We have conducted additional experiments and have now added two new main figures and four new supplementary figures.

•Figure 4 d and F, title of the graph says fluorescent intensity, but the graph indicates number of neurons, not intensities.
Fixed. Thanks

Second decision letter

MS ID#: DEVELOP/2024/202733

MS TITLE: UNC-30/PITX coordinates neurotransmitter identity with postsynaptic GABA receptor clustering

AUTHORS: Edgar Correa, Morgane Mialon, Melissa Cizeron, Jean-Louis Bessereau, Berangere Pinan-Lucarre, and Paschalis Kratsios
ARTICLE TYPE: Research Article

I am delighted to tell you that your manuscript has been accepted for publication in Development, pending our standard publication integrity checks. Referee reports on this version are appended below. You will see that Referee 3 has a few suggestions of minor changes that you may consider introducing in your final manuscript before forwarding it to our Editorial Office at dev@biologists.com.

Reviewer 2

Advance summary and potential significance to field

This revised study from Correa and colleagues addresses important questions about the relationship between presynaptic differentiation and postsynaptic organization. Specifically, the study investigates the coordination of neurotransmitter identity and postsynaptic receptor clustering through the actions of the UNC-30/Pitx terminal selector transcription factor in C. elegans GABAergic motor neurons. UNC-30 is required for the expression of genes that indicate GABAergic identity (unc-25/GADD, unc-47/vesicular GABA transporter etc).

The authors show here that UNC-30 also regulates the clustering of postsynaptic GABA A receptors through transcriptional control of the secreted synaptic organizer madd-4B, supporting a model for coordination between pre- and post-synaptic development through transcriptional mechanisms in the presynaptic neuron.

These findings closely align with previous findings from the authors’ labs supporting coordination of cholinergic identity and the clustering of postsynaptic receptors through the UNC-3 terminal selector. The present study extends this model for pre- and postsynaptic coordination by adding a number of additional interesting observations. The authors provide evidence that continuous

© 2024. Published by The Company of Biologists under the terms of the Creative Commons Attribution License (https://creativecommons.org/licenses/by/4.0/).

expression of unc-30 is required for the maintenance of GABA identity genes. The authors also show that UNC-30 also represses the expression of another madd-4 isoform involved in cholinergic synapse development, suggesting previously unrecognized dual transcriptional repressor/activator roles for UNC-30. Finally, the authors suggest additional targets for direct transcriptional activation and repression by UNC-30. These are significant advances. Overall, the manuscript is well-written, the experiments clearly performed and interpreted, and limitations acknowledged.

The additional experiments included in the revised version of the manuscript further strengthen the authors' conclusions.

Comments for the author

The authors have satisfactorily addressed my prior concerns. Congratulations on a very nice piece of work!

Reviewer 3

Advance summary and potential significance to field

The new version of the MS has additional data that strengthen the paper and address all suggestions

Comments for the author

I would like to congratulate authors for the extensive work they have performed addressing all reviewer's suggestions and comments.
I only have four minor suggestions
- Figure 8 I: please add the asterisk to continuous requirement in the model as in figure 7
- 459: "suggest a repressive role for UNC-30". I would add "likely indirect repressive role"
- line 277: typo previosuly
- Line 626: " It is likely that cooperation with distinct transcription factors shifts its transcriptional activity from an activator to a repressor". Despite unc-30 BS near madd-4L and glr-5 and unc-53, there is no evidence for functionality of those sites in repression (Fig S5D, no phenotype when site is mutated). Thus it would be advisable to be more cautious when saying "shifts its transcriptional activity from an activator to a repressor". I suggest:
"Cooperation with distinct transcription factors might shift UNC-30 transcriptional activity from an activator to a repressor, alternatively, UNC-30 might activate a TF that acts directly as repressor." Indeed, this incoherent feed forward loops have been previously described in C. elegans neurons subclass specification.

© 2024. Published by The Company of Biologists under the terms of the Creative Commons Attribution License (https://creativecommons.org/licenses/by/4.0/).
